# Overcoming hepatic tropism: Precision engineering of lipid nanoparticles for extrahepatic RNA delivery

**DOI:** 10.1016/j.mtbio.2026.103568

**Published:** 2026-08-18

**Authors:** Yu Liu, Xiao Guo, Qin Hu, Chunyuan Gan, Shili Nie, Jingheng Xiang, Yao Liu, Jie Zou, Xintong Wu, Chong Li, Yaqin Tang

**Affiliations:** a College of Pharmacy and Bioengineering, Chongqing University of Technology, Chongqing, 400054, People's Republic of China; b College of Pharmaceutical Sciences, Southwest University, Chongqing, 400715, People's Republic of China; c Chongqing Key Laboratory of Target-Based Drug Discovery and Research, Chongqing, 400715, People's Republic of China

**Keywords:** LNPs, Extrahepatic precision targeting, Protein corona engineering, Organ-selective delivery, Ionizable lipid design, Tissue-specific RNA therapeutics

## Abstract

Lipid nanoparticles (LNPs) are effective carriers for RNA and have become the standard of care; however, their natural affinity for the liver restricts their use elsewhere. To address this limitation, the “biological identity" of LNPs must be reprogrammed by modifying the three interconnected factors of lipid composition, physicochemical properties and protein corona modulation. Many previous studies have tended to evaluate individual regulatory factors in isolation; therefore, building on prior research, this review establishes a comprehensive conceptual framework that integrates these three dimensions to systematically elucidate the design principles for achieving extrahepatic targeting of LNPs. Targeting strategies are classified according to the mechanism of organ accumulation: (1) Endogenous corona-mediated (SORT, ENDO, POST), these approaches are independent of direct exogenous ligand–receptor interactions and rely on the recruitment of specific plasma proteins; within this category, POST is further distinguished by peptide-remodeled coronas; (2) Covalent active targeting - surface-conjugated ligands that directly bind to cell-surface receptors; and (3) Passive accumulation - regulated by vascular permeability. This review summarizes recent advances in delivery to the lungs, spleen, brain, heart, kidney, bone, and pancreas, discusses major translational bottlenecks (species differences, immunogenicity, and manufacturing), and proposes a direction for predictive design.

## Graphical abstract

Fig. 1 Engineering strategies designed to enable extrahepatic RNA delivery and overcome the inherent hepatophilicity of LNPs. Left: Intravenously injected LNPs (100–200 nm) accumulate in the liver via ApoE adsorption and LDLR-mediated hepatocyte uptake, while also being further cleared by Kupffer cells. Middle: Extrahepatic targeting can be achieved through composition modulation (lipid selection, pKa, particle size, zeta potential) and protein coat reprogramming reducing ApoE, enriching vitronectin (Vtn) and beta-2-glycoprotein I(β_2_-GPI), thereby directing the carrier to non-liver tissues. Bottom: Major barriers to translation include immunological challenges, species differences, scaling up production.

**Figure:**
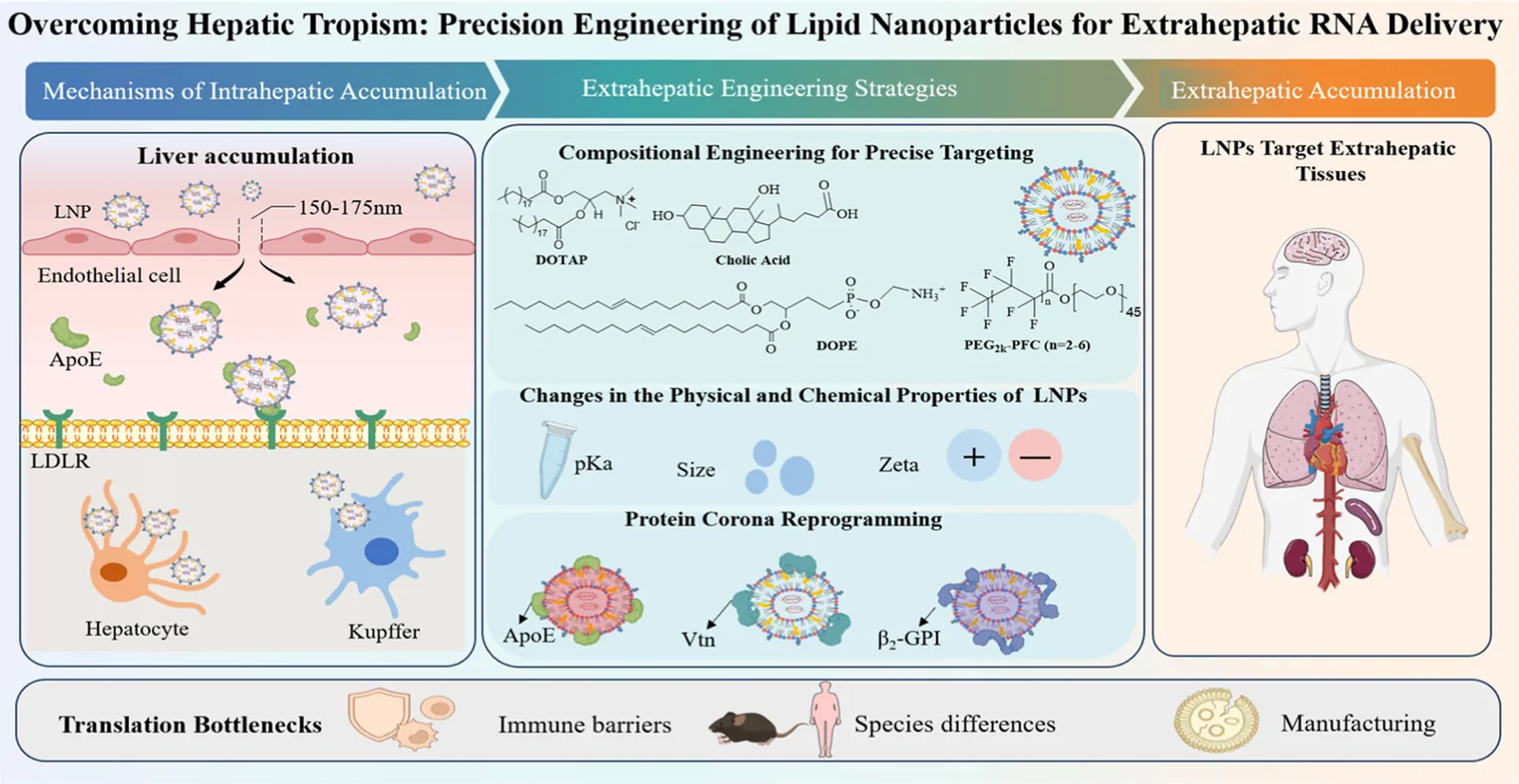

## 1 Introduction

In the past twenty years, various RNA-based therapeutics, including mRNA, siRNA and miRNA, have been developed and are now being applied in cancer treatment, genetic diseases, metabolic disorders and neurological diseases. Preclinical and clinical studies have shown that they hold promise for treating diseases that currently lack effective therapies [[1], [2], [3], [4], [5], [6]]. The convergence of RNA-based therapeutics and immunomodulatory agents has opened new avenues for durable and highly specific interventions [7,8].

Although RNA therapeutics have shown promise in preclinical and clinical studies, their widespread application remains limited by three major challenges: inherent instability, immunogenicity, and difficulties in achieving cell- and tissue-specific delivery [[9], [10], [11], [12]]. To address these barriers, LNPs have emerged as the leading non-viral delivery platform for nucleic acids, with the clinical authorization of mRNA-based COVID-19 vaccines marking a pivotal milestone [13]. Indeed, LNPs offer several advantages over other delivery systems, including favorable safety profiles and high transfection efficiency. However, a fundamental limitation of conventional LNPs is their intrinsic hepatotropism: following systemic administration, they rapidly associate with Apolipoprotein E (ApoE) and are internalized by hepatocytes via Low-Density Lipoprotein receptor (LDLR)-mediated endocytosis, resulting in predominant accumulation in the liver [[14], [15], [16], [17], [18]]. This hepatic bias severely restricts the application of LNPs-based RNA therapeutics to extrahepatic diseases and has motivated the development of strategies to reprogram LNPs tropism toward non-liver tissues [13,19,20].

**Fig. 1:**
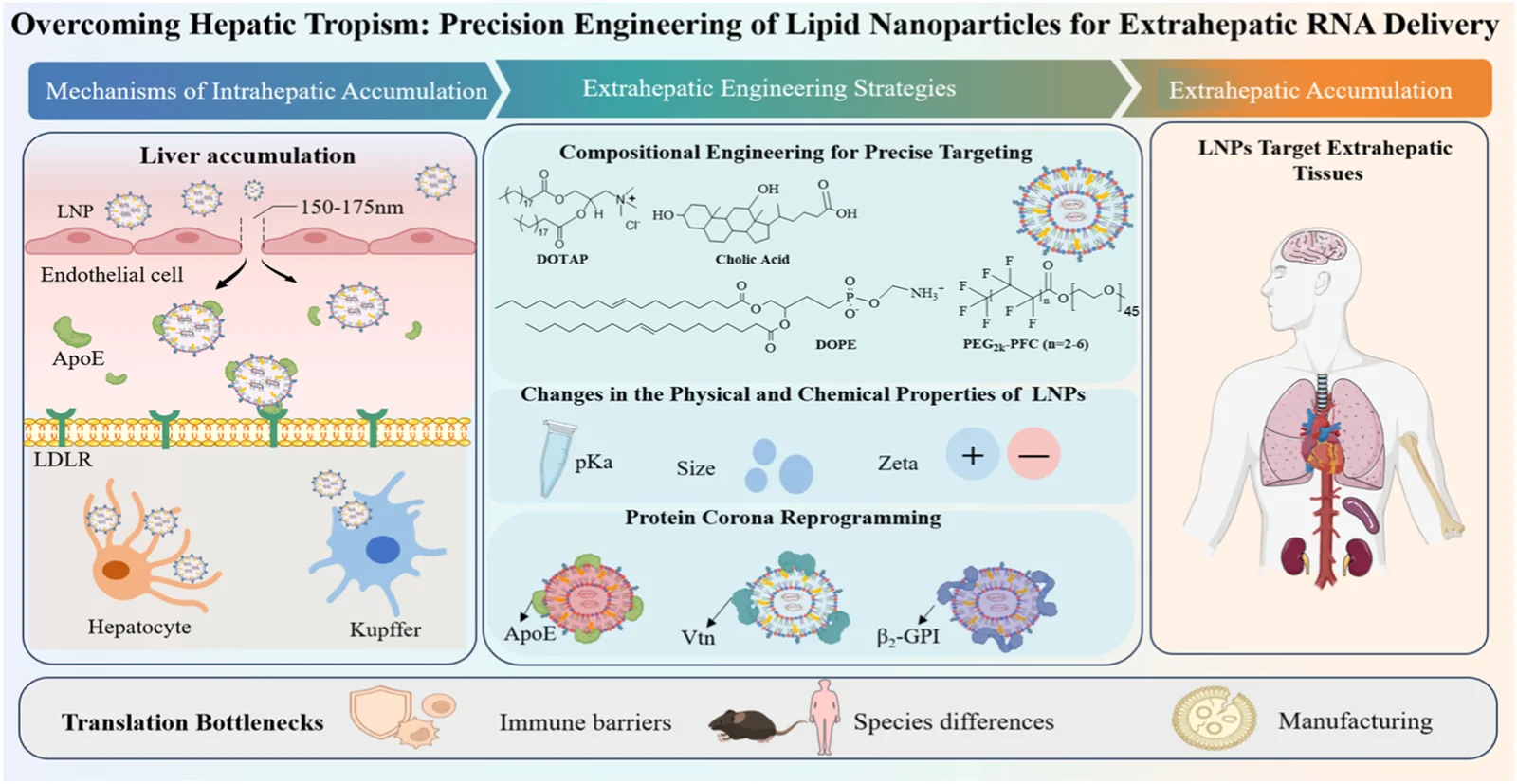
Engineering strategies designed to enable extrahepatic RNA delivery and overcome the inherent hepatic tropism of LNPs. Left: Intravenously injected LNPs (100–200 nm) accumulate in the liver via ApoE adsorption and LDLR-mediated hepatocyte uptake, while also being further cleared by Kupffer cells. Middle: Extrahepatic targeting can be achieved through composition modulation (lipid selection, pKa, particle size, zeta potential) and protein corona reprogramming reducing ApoE, enriching vitronectin (Vtn) and beta-2-glycoprotein I (β_2_-GPI), thereby directing the carrier to non-liver tissues. Bottom: Major barriers to translation include immunological challenges, species differences, scaling up production.

Recent reviews have summarized hepatic delivery, targeting of individual organs, or specific platforms such as SORT and POST. However, there remains a lack of a unified framework that integrates the three interdependent pillars governing LNP-mediated extrahepatic targeting—component engineering, physicochemical properties, and protein corona reprogramming. Moreover, the mechanistic interplay among these pillars and their combined impact on overcoming hepatic tropism have not yet been systematically analyzed. This review, centered on these three pillars, provides a systematic and comprehensive overview of the reprogramming of LNP biological properties from innate hepatic tropism to programmable extrahepatic specificity, critically analyzing the role of each pillar in this process. We also discuss the major translational bottlenecks (species barriers, immunogenicity upon repeated administration, and manufacturing challenges), propose a roadmap toward predictive design, and summarize the progress in targeted delivery to the lung, spleen, brain, bone, and heart, aiming to provide both a theoretical framework and practical guidelines for the development of tissue-specific nucleic acid-loaded LNP therapeutics (Fig. 1).

## 2 Composition of LNPs and physiological mechanisms of hepatic accumulation

### 2.1 Physiological mechanisms of hepatic accumulation

**Fig. 2:**
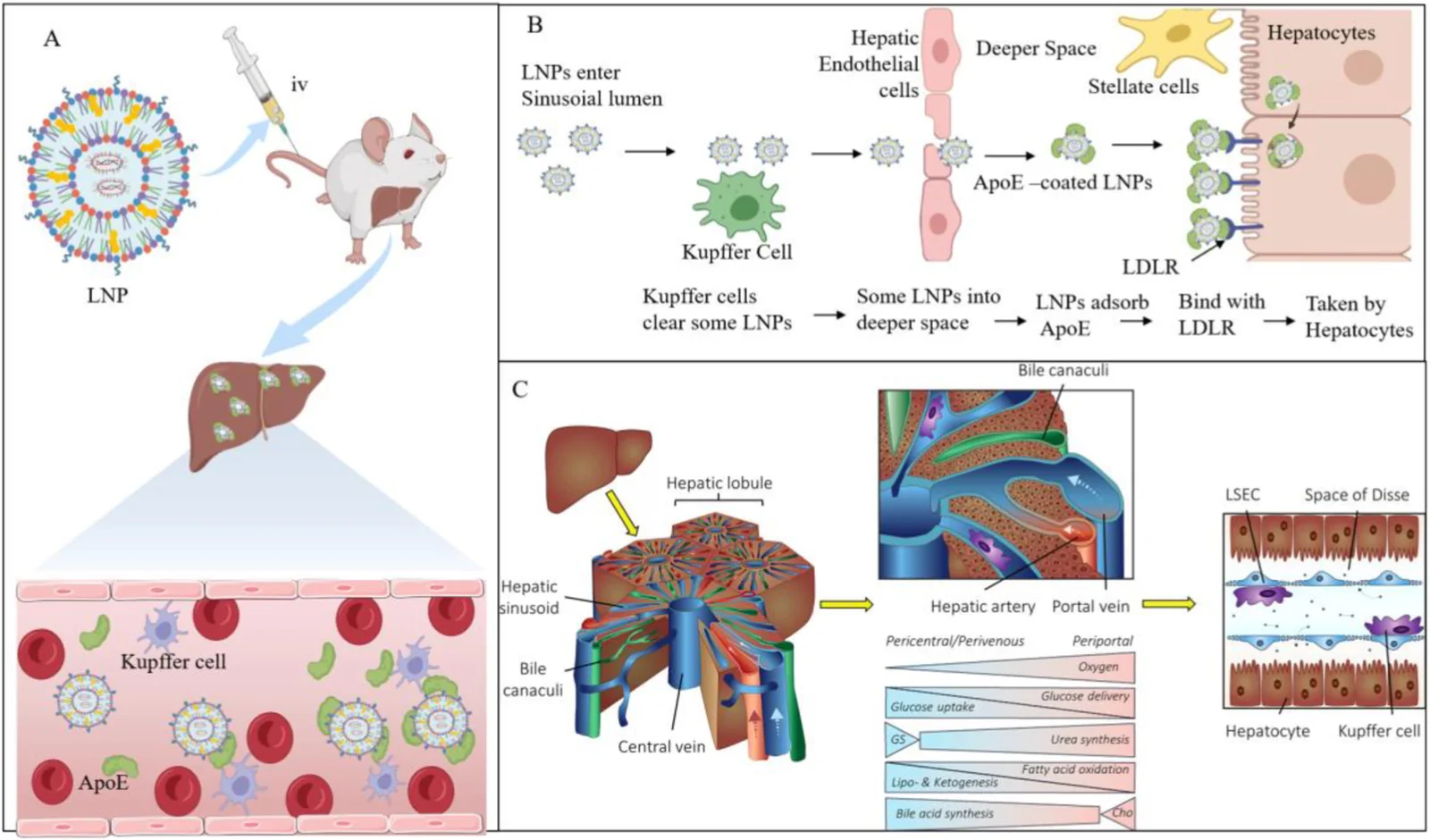
The physiological mechanism of LNP accumulation in the liver. A) After tail vein injection, circulating LNPs are coated with plasma ApoE, facilitating liver targeting; B) Circulating LNPs pass through the pores of the sinusoidal endothelial cells, are coated with ApoE in the disse space, and enter hepatocytes via LDLR-mediated endocytosis; C) Diagram of the structure of the liver lobule, showing the dual blood supply, the portal–central venous metabolic gradient, and the spatial distribution of sinusoidal endothelial cells, Kupffer cells, and hepatocytes; reproduced from Ref. [25] with permission from Elsevier, copyright 2020.

After injection into a vein or muscle, the blood-circulating LNPs rapidly bind to ApoE, a key apolipoprotein in the blood plasma [21]. In Fig. 2A, the adsorption of ApoE modifies the surface physicochemical properties of LNPs, allowing the nanoparticles to structurally and functionally mimic endogenous low-density lipoprotein (LDL) particles. This ApoE-enriched surface coating facilitates specific recognition by LDLR, ultimately promoting efficient hepatic uptake of LNPs [22]. ApoE strongly binds to LDLRs, which are highly expressed on the surface of liver cells. This binding event induces receptor-mediated endocytosis and thus preferentially localizes LNPs in the liver tissue [23] (Fig. 2B). This mechanism has been verified by experiments on murine models deficient in functional LDLR [24].

These knockout mice have significantly reduced hepatic LNP accumulation and thus indicate that the ApoE–LDLR pathway is required. Not all of the particle absorption occurs through receptor-mediated endocytosis, and adjustments in liver or blood flow can also promote this type of absorption. Fenestrations in sinusoidal endothelial cells (150-175 nm) can be used for extravasation and subsequent retention in the parenchymal space [26]. The functional hepatic lobules in the liver are supplied by a double-inflow system of the hepatic artery and portal vein, and these blood vessels eventually merge into central veins [25] (Fig. 2C). Hepatic sinusoidal endothelial cells and resident Kupffer cells on the inner wall of the hepatic sinusoids expose circulating LNPs to phagocytes. A high metabolic rate is required to quickly distribute LNPs, and extensive endothelial fenestration along with a slow sinusoidal blood flow extends the residence time of particles in the liver microvasculature [23,27]. Hepatic macrophages are biological filters that phagocytose LNPs via the mononuclear phagocyte system (MPS) and thus promote their accumulation in the liver [[28], [29], [30], [31]].

### 2.2 Fundamental components of LNPs: core elements and diverse innovations

The four main components of the typical LNPs are to encapsulate genetic material and promote targeted delivery at the site of disease. With the continuous development of this field, the molecular structure of the above components has gradually been elucidated, and new preparations have been developed to overcome the deficiencies of the traditional four-part system. Recently, some new developments have included streamlined single-chip architectures, tripartite formulations, and pentavalent assemblies. The expanded toolset can now customise the features of LNPs for all kinds of therapies more easily.

#### 2.2.1 The canonical four-component system of LNPs

**Fig. 3:**
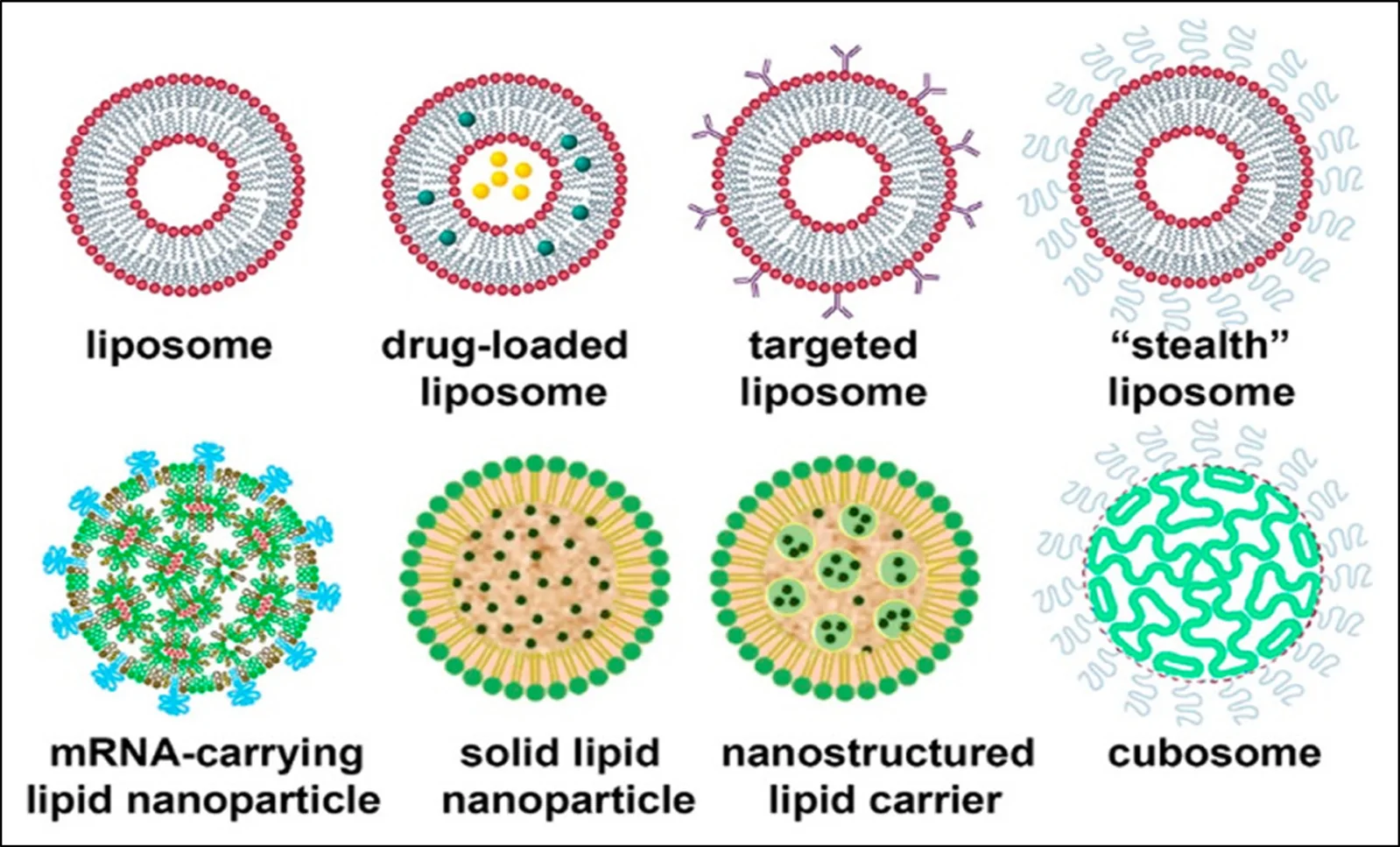
Developments in Lipid Nanomaterials. Overview of lipid-based nanocarriers, including liposomes, LNPs, and advanced formulations for biomedical delivery, reproduced from Ref. [35] with permission under CC-BY 4.0. Copyright 2021.

In general, the LNP system consists of four main components: ionizable lipids, helper lipids, and cholesterol, and polyethylene glycol (PEG) lipids. Each of these components controls physicochemical stability, maintains structural integrity, improves drug absorption, and governs biological behavior under experimental and physiological conditions [[32], [33], [34]]. As shown in Fig. 3, based on these basic components, various lipid delivery platforms have been developed, including liposomes, targeted liposomes, and LNPs loaded with mRNA; these platforms have a wide range of applications, from conventional drug delivery to nucleic acid therapy [35].

**Fig. 4:**
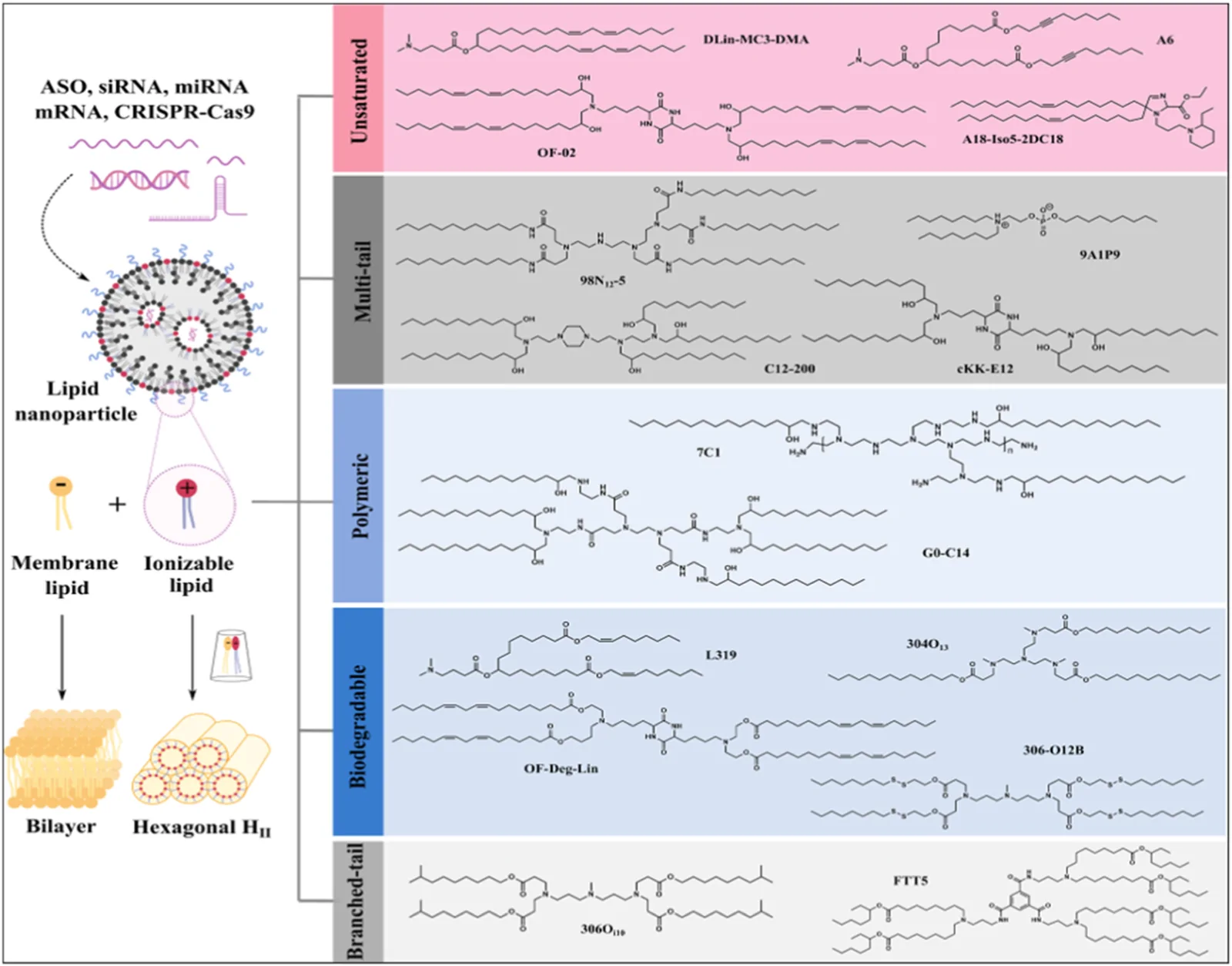
Structural and compositional basis for lipid nanoparticles to achieve endosome escape. Endosomal disruption mechanism: non-lamellar ionic complexes form between ionizable lipids and anionic phospholipids, enabling escape. Five structural classes: diglycerides (unsaturated lipids containing carbon–carbon double bonds), triglycerides (multi-tailed or amphiphilic lipids with more than two hydrophobic chains), polymers, biodegradable, and branched-tail, reproduced from Ref. [9] with permission under CC-BY 4.0. Copyright 2021.

Based on their structural features, lipids used for RNA delivery can be systematically categorized into five major classes: (1) diglycerides (unsaturated lipids containing carbon–carbon double bonds); (2) triglycerides (multi-tailed or amphiphilic lipids with more than two hydrophobic chains); (3) polymers (polymer-based systems with macromolecular or dendritic architectures); (4) biodegradable (lipids containing hydrolyzable ester, amide, or acetal bonds); and (5) branched (lipids whose hydrophobic regions evolve from a single scaffold to branched structures to modulate molecular geometry and enhance endosomal escape) (Fig. 4) [9].

However, conventional cationic lipids suffer from poor biodegradability and the potential for accumulation in tissues following repeated administration. To address these limitations, Ionizable lipids have gradually replaced traditional lipids. These lipids remain electrically neutral at physiological pH but become protonated and positively charged within acidic endosomes, thereby striking a balance between biosafety and endosomal escape capability [36]. Cleavable linkers, such as esters, amides and aldehydes, can be employed to facilitate the metabolic degradation and excretion of toxic substances. At the same time, MC3, SM-102 and ALC-0315 are widely used ionizable cationic lipids that reduce lipid aggregation and maintain transfection efficiency [37].

**Fig. 5:**
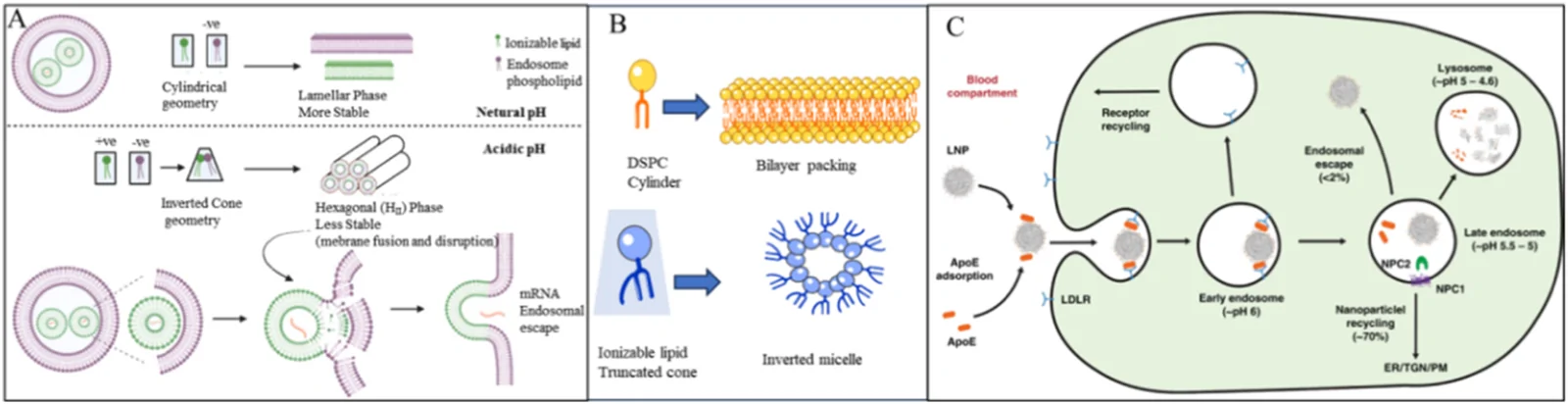
Behavior of LNP: pH-dependent lipid conformation, bilayer arrangement, and endocytosis A) pH-triggered LNPs transition: ionizable lipids protonate in endolysosomal acidity, interact with anionic lipids in endosomes, thereby inducing the formation of hexagonal phases and membrane disruption for cargo release, adapted from Ref. [38] with permission. Copyright 2024 National Academy of Sciences; B) Lipid bilayer theory: molecular geometry of amphiphilic compounds (e.g., DSPC, ionizable lipids) determines self-assembled structure, adapted from Ref. [39] with permission from Elsevier. Copyright 2022; C) Cellular uptake mechanism via PEG shedding, ApoE adsorption and LDLR-mediated endocytosis, adapted from Ref. [40] with permission under CC-BY 4.0. Copyright 2020.

Cationic polymers are based on the proton sponge effect to cause endosomal rupture; lipid nanoparticles, however, use a different method of lipid phase transition. Protonation of the ionizable lipid molecules is followed by association with endosomal phospholipids and intercalation of nonpolar hydrocarbon chains in the membrane bilayer. Thus, the first lamellar structure is converted into an inverted hexagonal array. The resulting structural rearrangement of the endosomal membrane allows the genetic material to enter the cytoplasm (Fig. 5A) [38].

Helper lipids account for 10-20 percent of the total content of LNP formulations and help maintain the structure of lipid nanoparticles for normal delivery. The molecular structure of the lipid affects the behaviour of LNPs in self-assembly and aggregation, and is therefore related to the functional attributes of the formulation (Fig. 5B) [39]. DSPC is a typical lipid carrier that has been optimized to a limited extent, and a good replacement with better delivery efficiency is needed. POPC-modified LNPs increased the translation efficiency of mRNA in hepatocytes by 2.49-fold, for example. Through this approach, phosphatidylcholine homeostasis is restored by delivering CCTα mRNA; thus, the accumulation of pathological lipid droplets is inhibited and the progression of metabolic dysfunction-associated fatty liver disease (MAFLD) is slowed, with good safety data [41]. This approach has built an ideal system for mRNA-based therapy of MAFLD and emphasized the need to target-screen helper lipids for the development of high-performance LNPs.

Helper lipids increase the density of the bilayer by hydrophobic interactions and thus prevent the leakage of nucleic acids in circulation. However, they do not directly trigger the phase transition required for endosomal escape; rather, protonated and ionizable lipids are mainly responsible for this function. Helper lipids regulate the system according to their molecular structures. DOPE has a short chain, thus it induces negative curvature and forms a hexagonal phase; consequently, it can be released from endosomes. On the other hand, due to its cylindrical molecular shape, DSPC does not participate in the phase transition and thus maintains the integrity of the lamellar bilayer. Both of the above architectural features and the choice of delivery vehicle directly affect the efficiency of transfection and delivery. In addition, LNPs can be modified to alter their interfacial properties and thus affect the fate and therapeutic results of adsorbed protein coronas and cellular engagement dynamics [42,43].

Cholesterol is required for the organisation and function of LNPs. Sterol molecules are located in the core of the phospholipid double layer and, by increasing the structural rigidity of the membrane, thus enhance its stability. Cholesterol can also be employed as a carrier for delivering drugs to target cells. Protection of the lipid carrier from damage by the MPS, enhanced uptake by cells, and shielding from harm caused by endogenous enzymes and other chemical substances [44].

High-cholesterol LNP formulations are closely associated with ApoE. Research by Patel has shown that phytosterols from nature, such as β-sitosterol and stigmasterol, can substitute for the C-24 position of sterols, and when these modified lipids (eLNPs) are used, both cell counting and improved mRNA retention, as well as enhanced transfection efficiency have been observed. This modified cholesterol significantly improved mRNA delivery, and its highly dispersed morphology was directly correlated with high delivery efficiency; In contrast, previously reported cholesterol-based LNPs are spherical and have low delivery efficiency. Furthermore, sterol-modified polyhedral LNPs possess stronger binding affinity for ApoE, which promotes LDLR-mediated cellular uptake, thereby enhancing mRNA delivery efficiency. This morphology-dependent ApoE affinity serves as the key link connecting sterol structural optimization with improved transfection performance (Fig. 5C) [40].

Although PEGylated lipids account for only 1.5%–2% of the total lipids in LNPs, they exert a significant regulatory effect on in vivo performance. The surface-hydrated PEG layer enhances colloidal stability, reduces clearance by mononuclear phagocytes, and prolongs circulation time, thereby supporting extrahepatic delivery [[45], [46], [47]].

However, this protective effect also gives rise to the well-known PEG dilemma. While PEG's shielding effect minimizes off-target internalization, it also hinders target binding and tissue penetration. Shorter acyl chains on the PEG lipids allow for gradual desorption from the surface in the bloodstream, which exposes the LNPs' surface to bind ApoE and initiates the uptake process via the LDLR in the liver. Excessively long PEG chains can limit the formation of a functional protein corona and reduce targeting efficiency. Therefore, optimal LNPs design requires a delicate balance between circulatory persistence and timely surface exposure, which can be achieved by modulating the chain length, molecular weight, and ratio of PEG-conjugated lipids [45].

In addition, repeated administration of PEGylated LNPs often leads to accelerated clearance from the bloodstream and triggers adverse immune reactions. Such adverse reactions can be mitigated through advanced structural optimization and stealth surface engineering of DSPE-mPEG derivatives [48]. In combination with hyaluronic acid modification, these nanoparticles are also able to target tumors specifically and thus deliver the drugs more precisely to the diseased sites [49].

#### 2.2.2 Component Innovation: From Simplification to Functional Diversification

In addition to the four parts of the original LNPs, researchers have also improved their delivery performance by altering the components, adding auxiliary substances, excluding certain components, etc., to enhance targeting specificity and efficiency of gene delivery.

**Fig. 6:**
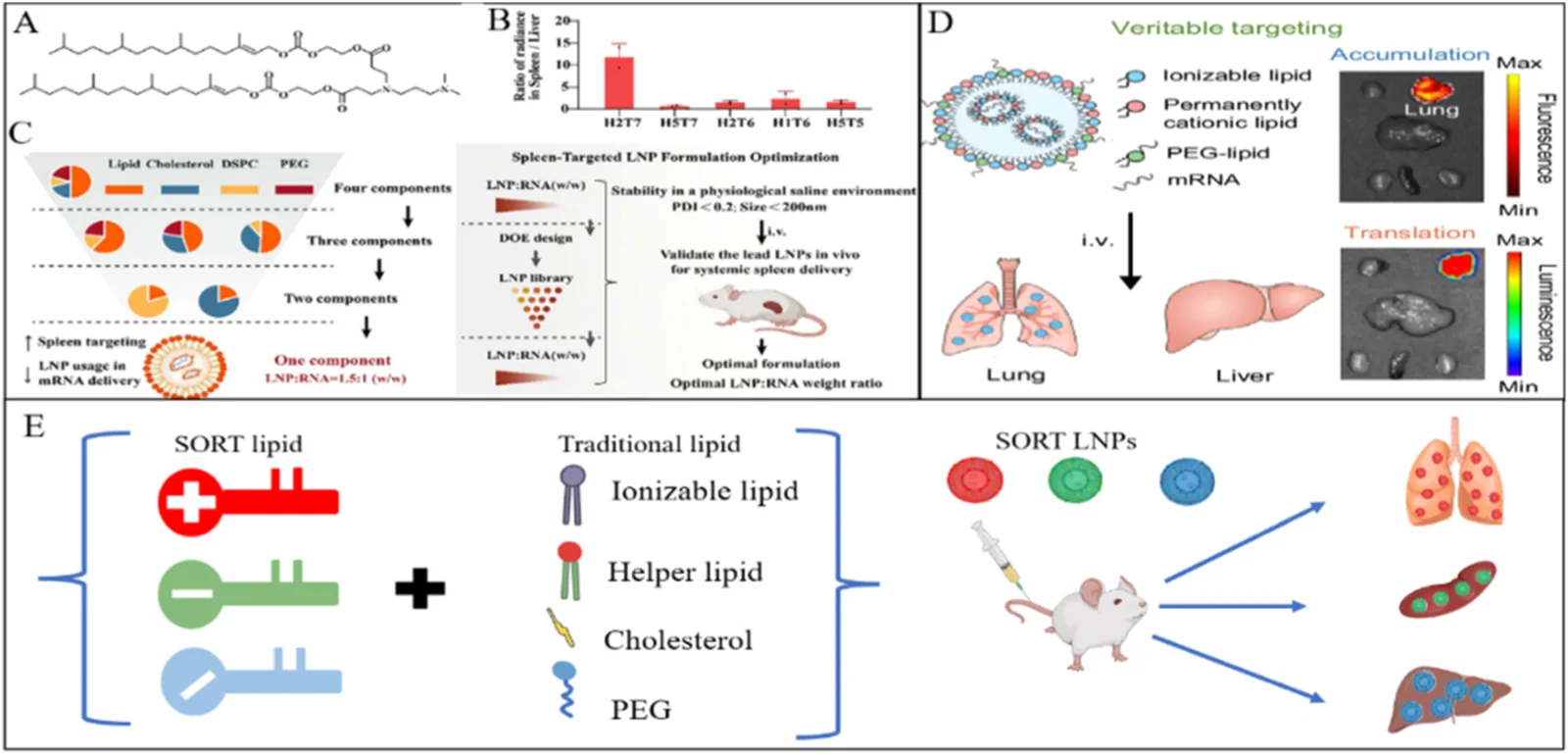
Component Innovation: From Simplification to Functional Diversification. A) Chemical structure of H2T7. B) DMA-Liposome mRNA Expression Ratio in Spleen and Liver. C) Iterative screening workflow identifying OncoLRC as a splenic-homing mRNA delivery vector; A-C adapted from Ref. [51] with permission under CC-BY. Copyright 2025. D) Dual optimization of lipid composition and architecture yielded 6Ac1-C12 Liver LNPs with hepatic tropism, shown by mRNA accumulation and protein expression, adapted from Ref. [53] permission under CC-BY 4.0. Copyright 2024; E) siRNA SORT LNP formulations with five lipid components. Specific SORT lipids enabled targeted delivery: DOTAP-50 (lung/kidney), DODAP-20 (liver), and 18 PA-15 (spleen) in murine models.

Monodisperse LNPs are not surfactants, cholesterol or PEGylated lipids. Although they are able to selectively target dendritic cells and other APCs, T cells have structural deficiencies due to a rigid lipid composition and are not clinically applicable. Prof. Shuai Liu [50] devised monomeric ionizable cationic lipids that greatly boost targeted mRNA delivery into splenic tissue and T lymphocyte subsets. After intravenous injection, this formulation effectively transfects diverse T cell populations, highlighting its strong potential for novel immunotherapies. In the single-component arena, Min Qiu [51] engineered OncoLRC, an optimized lipid–mRNA complex formed from a single lipid species with splenic tropism. Systemic administration selectively delivers mRNA to splenic APCs. Systematic screening yielded a lead dimethylamino (DMA)-based formulation that provides an optimized lipid coating, eliminating the need for conventional four-part LNP designs (Fig. 6A–C). A single-structured cationic micelle (DMP) system based on lipid/block copolymer compositions has also been developed for efficient mRNA delivery in cancer immunogene therapy [52].

The ternary LNPs structure achieves an optimal balance between structural simplicity and targeting diversity. Liu and colleagues [53] designed a cholesterol-free ternary LNP that overcame passive liver uptake and enabled pulmonary mRNA delivery, providing a practical strategy for precision mRNA therapy (Fig. 6D). In addition, at the moment this team has developed phospholipid alkylated polymers (PAPs) through low-cost commercial cationic polymer modification: moncomponent PAPs can deliver mRNA specifically to the spleen, and the efficacy of the vaccine following intravenous administration has been validated in a mouse melanoma model; when combined with helper lipids to form four-component LNPs, PAPs can achieve specific mRNA delivery targeting the lungs and liver, respectively [54]. Zhang et al. [55] designed ternary Tc-LNPs incorporating ionizable cholesterol lipids. Conjugation of the cholesterol backbone with pH-responsive ionizable groups reduces ApoE binding, thereby preventing liver accumulation via the LDLR pathway and increasing spleen targeting. The ISL-3C-LNPs, which also exhibited superior mRNA encapsulation and transfection performance, were further developed. This improved formulation based on CS22021 limits systemic dissemination by retaining locally in the muscle, provided improved biosafety and elicited a strong immune response when used as an mRNA vaccine platform [56].

As a strategy to enhance efficacy, SORT LNPs complements the traditional four-component system by incorporating specific functional lipids, thereby enabling flexible biological targeting. This platform achieves sustained, dose-dependent gene silencing in the lungs, liver, and kidneys; following repeated administration, protein levels in lung tissue can be reduced by up to 88% (Fig. 6E) [57].

Compared to simplified designs, the five-component LNPs achieves precise control over organ tropism by increasing formulation complexity. Li and colleagues introduced an ionizable diamine cholesterol analog as the fifth functional component into a commercial LNPs. By precisely adjusting the proportion of this fifth lipid, organ-specific targeting can be achieved: the optimized formulation achieved mRNA delivery efficiencies of 95% and 78% in the spleen and lungs, respectively [58].

## 3 Extrahepatic precision targeting strategies: overcoming “liver preference”

### 3.1 Three key factors influencing the fate of LNPs in vivo: composition, physicochemical properties, and protein corona

The biological identity of an LNP emerges from the interplay of three interdependent elements, not from any single factor. These components, the lipid core, the physicochemical properties, and the protein corona, constitute an inseparable system. A holistic approach is therefore needed to reprogram LNP properties for extrahepatic targeting, one that accounts for these interrelationships rather than isolating each variable. The following sections of this paper will discuss the above three basic factors in detail and explore how to use current platforms to realise all-round development.

### 3.2 Compositional engineering for precise targeting: Optimization of Ionizable Lipids

#### 3.2.1 Ionizable lipid optimization

**Fig. 7:**
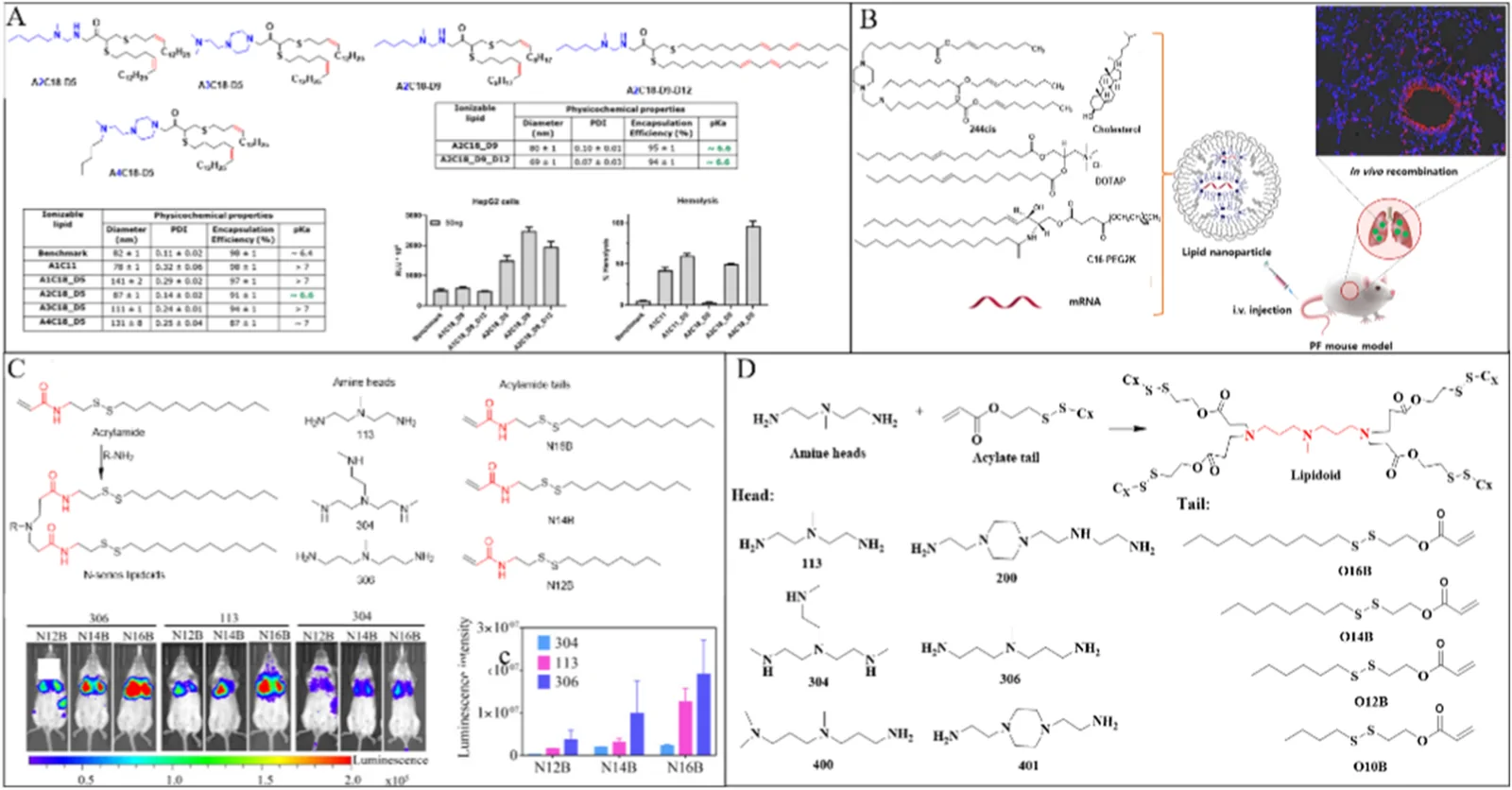
Optimization of Ionizable Lipids. A) the chemical composition of sulfide A1C11; the physicochemical properties of mRNA-delivering liposome nanoparticles prepared using A1C11 and various helper lipids (DSPC, DOPE); and the luciferase activity expressed in HepG2 and C2C12 cells in different LNP formulations, adapted from Ref. [60] with permission under CC-BY 4.0. Copyright 2025; B) Piperazine-modified lipid derivatives accumulate in the lungs, adapted from Ref. [61] with permission under CC-BY. Copyright 2023; C) Synthesis pathways and representative chemical structures of lipids, as well as representative whole-body bioluminescence images of mice obtained using the IVIS imaging system and the in vivo mRNA delivery efficiency of the N-series LNPs, adapted from Ref. [62] with permission from PNAS. Copyright 2022; D) Chemical structure of tail-branched biodegradable lipids, adapted from Ref. [63] with permission from PNAS. Copyright 2021.

Molecular architecture of ionizable lipids is required to ensure the efficiency of LNPs delivery [59], but structural modification efforts are still facing significant practical limitations. According to Gary Davidson's research, when ionizable lipids with unsaturated alkyl chains are combined with highly lipophilic amino acid heads, the expression level of intracellular proteins is enhanced, haemolytic toxicity is reduced, and the efficiency of systemic mRNA delivery is increased by more than 200 times (Fig. 7A) [60]. However, too much hydrophobic modification can lead to lipid aggregation and reduced LNPs stability in physiological conditions, thereby restricting their clinical translation.

Modifying the structure of ionizable lipids through headgroup chemistry, tail engineering, or linker optimization can substantially alter LNPs biodistribution and targeting. However, such changes often lead to high selectivity for a single organ. Lipids bearing imidazole-modified head groups, or IMILs, can enhance splenic targeting by up to 18.3-fold [64]. A comparative study of polyaminoethyl-modified ketone lipids identified BAMPA-O16B as the optimal lipid; its ideal pKa value allows mRNA LNPs to effectively escape from endosomes in the glioblastoma model [65]. Conversely, piperazine-modified lipid derivatives showed significant enrichment in the lung tissue (Fig. 7B). [61]. Additionally, Xu et al. [66]. varied the methylene spacer length between the amine headgroup and the ester bond in ionizable lipids. A change of a single methylene unit shifted the apparent pKa from 6.79 to 7.33, and the best performance occurred at a pKa of about 6.9. The mRNA delivery efficiency of this method is higher than that of the clinical benchmark drug SM-102. These findings collectively highlight the importance of precise design of the head group in the design of ionizable lipids.

In summary, by making small, precise changes to lipid structure, one can significantly alter the distribution of LNPs in tissues. However, current structural optimization strategies can only achieve significant improvements in a single system, and there is no easy or controllable way to target multiple systems; Thus, the diversity of LNP delivery systems and their broad use in therapeutic applications is severely constrained.

In addition to the polar head structure, the physicochemical properties of the bonds linking the polar head to the hydrophobic lipid tail are the primary molecular factors determining the biodegradability behavior of LNPs. As an illustration, the rational introduction of enzyme-sensitive ether group enables specific hydrolysis in the endosomal lumen, thereby facilitating the targeted transport of degraded lipid fragments to the lung tissue [67]. In contrast, evidence indicates that amide and urea bonds enhance degradation kinetics within pulmonary tissue. Accordingly, the nonpolar alkyl tail region of surfactants plays a crucial role in their bioactivity; the majority of these surface-active molecules possess between one and four lipophilic chains, with each chain generally comprising 8 to 20 carbon atoms [67,68]. These lipophilic tails may have either symmetrical or asymmetric alkyl arrangements. Among the above structural features, alterations in the three-dimensional shape of the membrane are the main causes of changes in membrane fluidity and nanostructure stability. These physicochemical characteristics will affect the distribution in the body and thus the efficacy of gene-cargo delivery [69]. From this standpoint, emerging investigations have demonstrated that alterations to lipid tail structures can function as design variables for directing mRNA preferentially to particular organs, thus facilitating accurate genome editing within intended tissues in living organisms via CRISPR-Cas9 systems [70]. Furthermore, the molecular pathway for degradation dictates the end point of these altered molecules: N-chain structures with amide-linked side groups are more likely to spread systemically (Fig. 7C) [62], whereas O-chain architectures containing ester groups are mainly retained in the liver (Fig. 7D) [63]. Symmetrically branched extended alkyl configurations enhance mRNA expression in the liver, while short-chain branched tails redirect gene-editing transcripts to splenic sites [71]. A conical molecular structure can also be achieved by adding polymeric segments to the lipophilic tail; such a structure is more readily incorporated into the liposomal bilayer and therefore more efficient at releasing DNA in the cytoplasm [72].

Recently, the first examples of advanced artificial intelligence platforms, such as AGILE (AI-Guided Ionizable Lipid Engineering), have appeared outside of traditional empirical selection. Combinatorial synthesis methods and neural network-assisted computational screening have been employed to speed up the discovery of ionizable lipids, and cell-type-specific lipid preferences have been identified for personalised LNPs design in various therapeutic applications [73]. LUMIlab is one such AI-powered platform that has been developed based on traditional high-throughput screening to accelerate the discovery of ionizable lipids. By combining predictions generated by foundation models with automated synthesis and screening, this platform can explore a vast region of chemical space that is difficult to reach through experimental screening alone and thus represents a new model of intelligent design [74].

#### 3.2.2 Cholesterol substitution

**Fig. 8:**
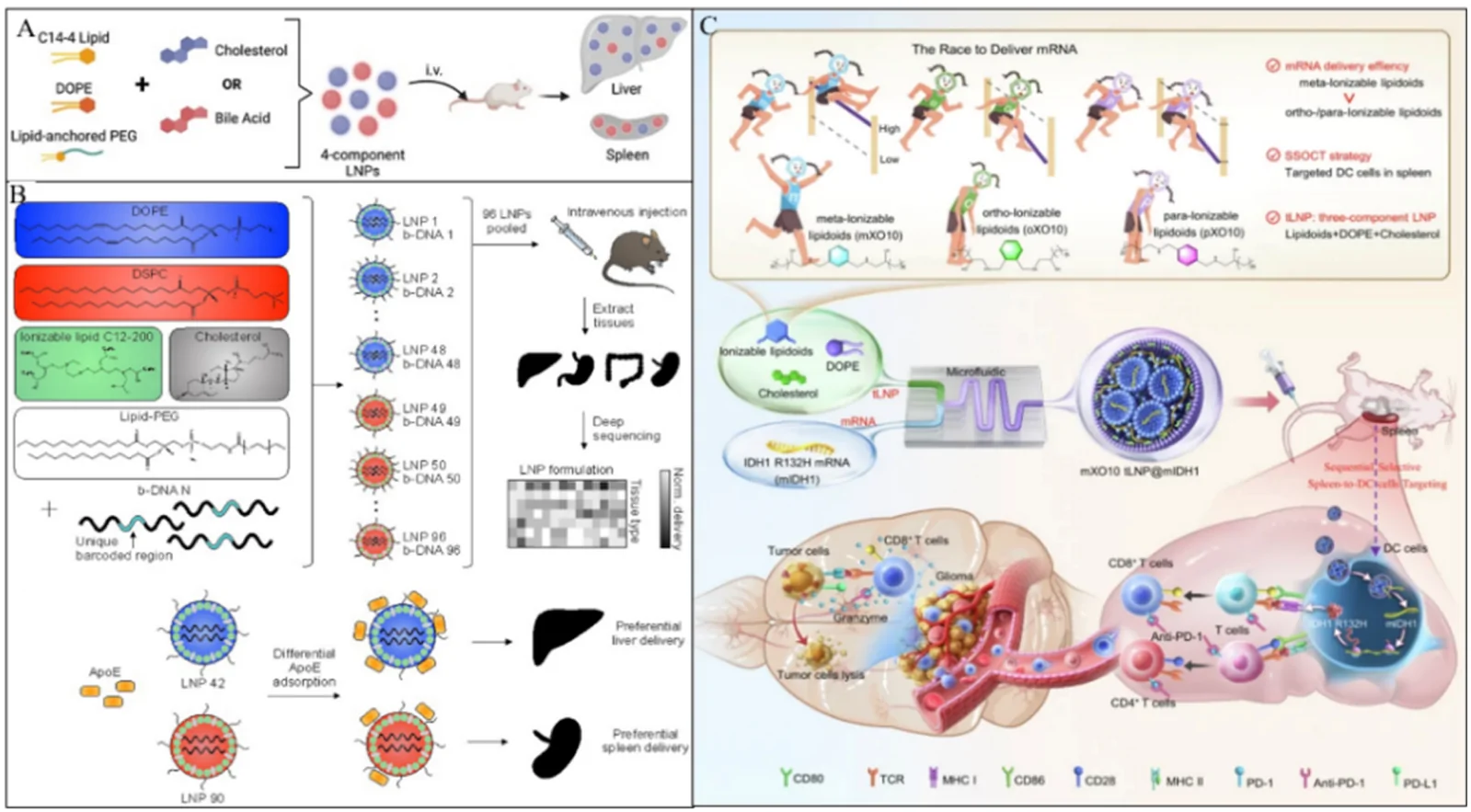
Engineering design of components for targeted LNPs platform. A) C14-4lipi target spleen, reproduced from Ref. [75] with permission under CC-BY 4.0. Copyright 2024; B) large-scale screening screening of 96 LNP formulations via in vivo sequencing analysis and use QCM-D detection of LNP-ApoE affinity and liver/spleen distribution, reproduced from Ref. [77] with permission from the Royal Society of Chemistry; C) Positional isomer-based ternary LNPs for splenic dendritic cell targeting and combined glioma immunotherapy, reproduced from Ref. [78] with permission under CC-BY 4.0. Copyright 2025.

Mitchell [75] investigated the use of bile acid derivatives (cholic acid, chenodeoxycholic acid, deoxycholic acid, or lithocholic acid) as cholesterol substitutes, conducting a systemic study using different molar ratios, and ultimately found that C14-4lipid exhibits spleen targeting (Fig. 8A). Separately, Bhattacharya and colleagues [76] pioneered a new class of LNPs called cortico-LNPs, in which endogenous corticosteroids were used to replace traditional cholesterol, thereby successfully redirecting the systemic biodistribution profile from the liver to the spleen.

#### 3.2.3 Helper lipid modulation

Helper lipids are not merely structural stabilizers but active regulators of LNPs tropism. In addition to providing passive structural support, chaperone lipids are capable of dose-dependently regulating protein corona formation, thereby actively modulating the organotropism of LNPs - an aspect that has long been overlooked in traditional LNPs design. An increase in phospholipid levels improves ApoE adsorption, thereby favoring transport to the liver; however, this simultaneously weakens binding to Vtn and causes a shift in transport towards the liver, thereby compromising targeting specificity to the lungs and spleen [41,79,80]. QCM-D-based validation further confirmed that DOPE-rich LNPs exhibit stronger affinity for ApoE, thereby achieving liver targeting; in contrast, DSPC-based LNPs weaken ApoE binding and promote spleen accumulation (Fig. 8B) [77].

In short, helper lipids serve not only as stabilizers for LNPs but also as key regulators of their targeting properties. Current optimization efforts rely primarily on empirical screening and lack quantitative, standardized design principles, which limits the precision and universality of advanced LNPs therapeutic systems.

#### 3.2.4 PEG optimization

Conventional PEGylated LNPs inevitably face the PEG dilemma, including impaired tissue penetration and potential PEG-related immune allergic responses, which limit their targeted delivery performance. To address these limitations, Cen et al. [81] synthesized fluorinated PEGylated LNPs via ultrasonic treatment, achieving a fourfold increase in splenic mRNA transfection compared to conventional formulations. To completely eliminate PEG-associated adverse effects, Deng et al. developed PEG-free ternary LNPs based on phenylenediamine-derived ionizable lipids. The optimal meta-isomer formulation enables precise mRNA delivery to thoracic tissues and splenic dendritic cells (Fig. 8C) [78]. However, PEG-free LNPs generally exhibit inferior circulatory stability, and their scalable clinical application still requires further systematic validation.

### 3.3 Core mechanism: synergistic effects of protein corona and physicochemical properties

Targeting tissues other than the liver with LNPs requires the coordinated action of many factors and is not due to any single reason. Protein corona formation in vivo and the intrinsic physicochemical properties of particles work together to regulate biological recognition and distribution patterns in tissues, as well as homing efficiency [82]. pKa, surface charge and particle size are relatively small parameters that affect cellular uptake and systemic bioavailability; therefore, they must be optimized to promote effective extrahepatic delivery [[83], [84], [85]].

#### 3.3.1 Protein corona: A “biological Label" for targeted recognition

##### 3.3.1.1 Composition and characteristics of the protein corona and its influencing factors

Traditional active targeting frequently underperforms in vivo compared with endogenous targeting strategies. Mechanistically, this disparity arises from three factors: (1) the protein corona rapidly coats ligand-modified LNPs, physically occluding targeting motifs and interfering with ligand–receptor engagement [86]; (2) exogenous ligands (antibodies or peptides) are recognized as foreign motifs and captured by the MPS in the liver, which accelerates LNPs clearance [87]; and (3) the dense PEG layer can sterically hinder ligand–receptor interactions, a phenomenon often referred to as the “PEG dilemma." In contrast, endogenous targeting—exemplified by SORT, POST, and endogenous targeting platforms (ENDO)—modulates intrinsic LNPs properties to selectively recruit endogenous opsonins [e.g., Vtn, β_2_-GPI, or Vitamin D Receptor (VDR)]. The resulting protein coronas engage conserved receptor pathways (e.g., αvβ_3_ integrin or CD169) and have been shown to improve extrahepatic specificity in various preclinical models.

In 2007, Cedervall et al. [88] first proposed the concept of the protein corona, a dynamic protein layer spontaneously assembling on nanoparticles in biological fluids. The protein corona is central to cell recognition and the in vivo performance of LNP formulations. Far from being a passive shell, it acts as a dynamic transducer that translates LNPs physicochemical properties into biological responses. The adsorption of plasma proteins is determined by properties including surface charge, pKa, and size. Once formed, the corona can direct organ tropism through receptor binding on target cells, a mechanism common to multiple targeting platforms. In this way, the corona connects LNPs design to extrahepatic specificity through a causal chain: physicochemical changes remodel the corona, which in turn alters tissue targeting [83,89,90].

Protein corona formation is influenced by characteristics of nanoparticles, such as size, charge, shape, and PEGylation, as well as the external biological environment. The surrounding fluid is also relatively high in ApoE; thus, it promotes increased adsorption of ApoE-rich lipoproteins compared with plasma and improves cellular uptake. In addition, the components of LNPs, such as size, charge, pKa, and PEG density, determine the structural framework that regulates in vivo targeting and delivery [91].

The scale of the particles determines where these living beings will be in the body. LNPs with a particle size of less than 50 nm are more likely to cross the blood-brain barrier, and those larger than 100 nm are more easily cleared by the liver [92]. Therefore, the present knowledge of this problem is still deficient in both theory and practice. Previous studies have only shown that LNPs properties/biological environments are correlated with modifications in the protein corona, but have not built an all-encompassing, quantitative prediction model. The dynamic, real-time growth of the protein corona in the bloodstream and its in vivo stability have not been fully investigated either. These deficiencies prevent precise, programmable control of the structure of the protein corona and thus reduce the capacity for intelligent design of a high-accuracy, targeted LNP delivery system.

LNP has a pKa value, so it will be in its charged or uncharged state at physiological pH and thus affect its ability to interact with cell membranes. An intermediate pKa in the range of 6.2–7.0 can be used to optimise release from endosomes and reduce non-specific binding to anionic cell membranes and serum components; thus, it is less likely to be cleared off-target or cause systemic toxicity. Surface charge affects lung adhesion and organ tropism; positively charged LNPs are more likely to accumulate in the lungs but have the risk of thrombosis and are thus limited in clinical application [93].

**Fig. 9:**
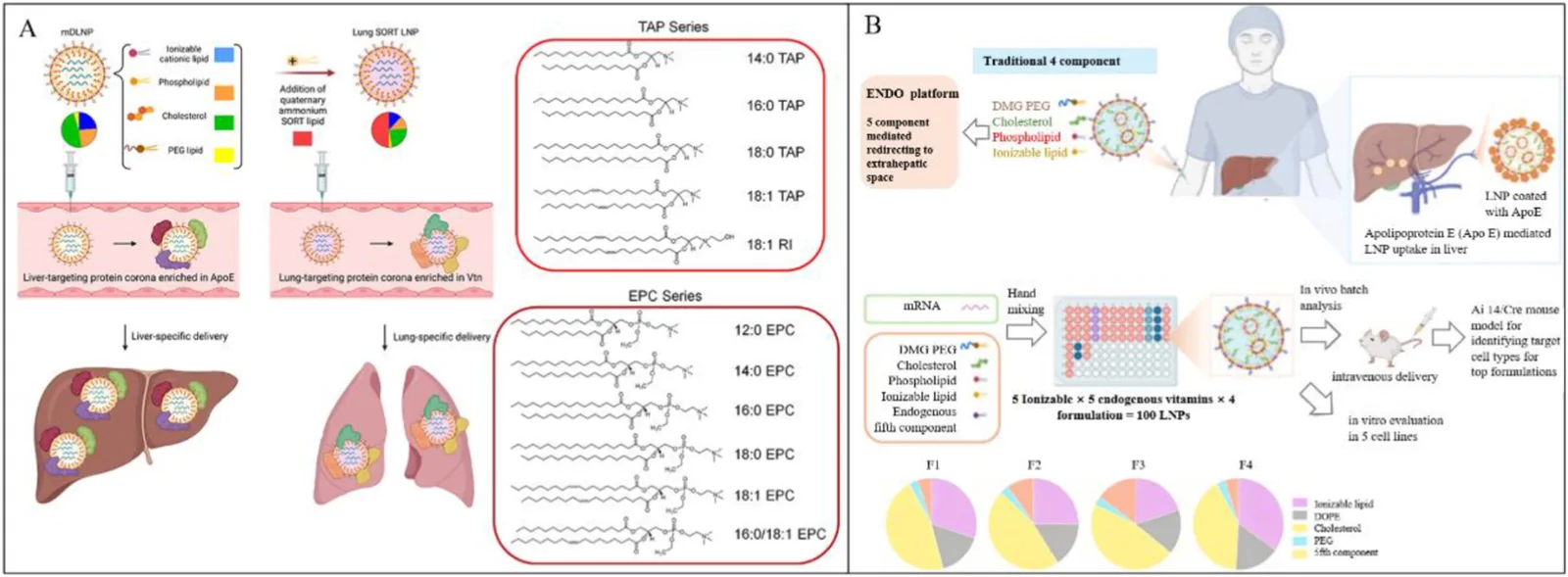
The SORT and ENDO platforms for LNP-mediated organ targeting using endogenous protein capsids. A) Conventional LNPs target the liver via the ApoE coat; SORT lipids redirect targeting to the lungs by promoting the formation of a Vtn-rich coat, along with the structures of the tested TAP and EPC series of SORT lipids, reproduced from Ref. [94] with permission from Elsevier. Copyright 2023; B)Pie charts depicting the compositional ratios of ionizable lipid, phospholipid, cholesterol, PEG lipid, and the endogenous vitamin as the fifth component, reproduced from Ref. [95] with permission under CC-BY.

In addition to passively influencing biodistribution, proper protein engineering enables dual control over targeted delivery and the immune response. As shown in Fig. 9A, by altering the physicochemical properties of LNPs (for example, by introducing SORT lipids to change the surface charge) it is possible to selectively recruit endogenous plasma proteins: conventional liver-targeted LNPs are rich in an ApoE protein corona, while lung-targeted SORT-targeted LNPs preferentially bind to Vtn, enabling the formation of a custom coating profile to achieve precise endogenous targeting [94]. The emerging POST strategy represents a primed variant of endogenous corona targeting. Unlike canonical covalent active targeting ligands that directly engage cell-surface receptors, the covalently tethered short peptides on POST LNPs act only as surface modulators to alter plasma protein adsorption kinetics and corona composition in circulation. Specific peptide motifs (6R for lung, 6D for spleen) remodel the protein corona to enrich endogenous organ-tropic proteins (Vtn, β_2_-GPI, tissue-specific secreted glycoproteins), which execute the ultimate tissue recognition and uptake process. This peptide-mediated corona reprogramming is not a new type of active targeting; instead, the conjugated ligands act as the recognition elements and do not regulate the corona [14].

Although there have been some recent attempts, the construction of the protein corona is still challenging. One such problem is how to achieve a stable balance between homing specificity and immune-regulatory functions. Another problem is that a minor change in the environment of surrounding cells can lead to an irregular change in the corona's structure, thus reducing targeting efficiency and provoking an allosteric alteration of the immune system. In addition, the POST platform is still in the exploration stage and lacks sufficient in vivo verification and extended-term safety studies to support large-scale translation.

##### 3.3.1.2 Targeted regulation function: specific components influence the composition of the protein corona to determining organ tropism

As shown above, the first type of LNPs typically uses ApoE binding and LDLR-mediated uptake for accumulation in the liver [96,97]. However, a single liver-targeting characteristic is no longer adequate to meet all the different demands of clinical practice.

The SORT strategy enables simple and scalable reprogramming of LNPs organ targeting by incorporating charged lipids as a fifth formulation component. This simple, modification-free modulation method alters surface charge and protein-coating characteristics, thereby enabling precise extrahepatic targeting: cationic and quaternary ammonium lipids (e.g., DOTAP, 18:1 TAP) confer lung tropism, while anionic lipids (e.g., 18 PA) redirect LNPs to the spleen. As a versatile platform, SORT-LNPs support tissue-specific mRNA expression and siRNA silencing in various non-liver tissues [98,99]. More in-depth mechanistic studies have shown that subtle structural differences in quaternary amine lipids—such as 18:1 RI headgroup modification—can alter the composition of the protein corona, particularly the concentration of Vtn, thereby enhancing the efficacy of lung targeting [100]. However, this strategy lacks standardized design rules and cannot precisely tailor organ tropism for different therapeutic scenarios.

Recent studies have shown that SORT-LNPs have organ tropism by forming different protein coronas. Vtn-rich LNPs target the lungs by binding to integrin αvβ_3_ on pulmonary endothelial cells. Conversely, β_2_-GPI-coated LNPs are recognized and captured by CD169^+^ macrophages in the spleen, thereby evading immune clearance [[101], [102], [103], [104], [105], [106]]. Although the core targeting mechanisms have been elucidated, most studies continue to focus on the static functions of proteins. The dynamic evolution of protein capsids in vivo and how formulation parameters modulate protein adsorption require further investigation.

Shao et al. developed the POST strategy, which achieves programmable extrahepatic delivery via conjugating short peptides onto the surface of LNPs [14]. Peptide sequence and length modulate protein corona formation and thereby determine organ selectivity. LNPs bearing 6R modifications predominantly localize within pulmonary tissue, whereas their 6D-modified counterparts home to the spleen through synergistic protein associations. This strategy proves compatible with diverse lipid scaffolds, and even minor peptide alterations can drastically reshape the ensuing targeting pattern. To date, only a small number of hydrophilic short peptides have been screened in this field, and the intrinsic relationship between peptide structure and the protein corona remains to be elucidated.

Siegwart's team [107] developed LNPs capable of recruiting Vtn for the targeted treatment of clear cell renal cell carcinoma (ccRCC). By binding to the αvβ_3_ integrin, which is overexpressed in tumors, this system significantly improved mRNA delivery efficiency both in vitro and in vivo. Bhattacharya developed the ENDO platform by adding vitamin D3 to the standard LNPs formulation, achieving over 99% selectivity. This platform demonstrated its ability to perform pancreatic-targeted gene editing in the Ai14 transgenic mouse model, achieving high-level td-Tomato expression in β-cells. These findings underscore the platform's potential for protein replacement therapy and CRISPR/Cas9-mediated gene editing, and offering new therapeutic opportunities for diseases such as pancreatic cancer and diabetes, which currently lack curative treatments (Fig. 9C) [95]. Mechanistic studies of the vitamin D3-based ENDO platform have revealed a unique corona-mediated mechanism of pancreatic targeting [[108], [109], [110]]. As the primary functional component of C-CholF3LNP, vitamin D3 recruits circulating, non-mobile VDR to assemble a specific coronal protein system on the LNPs surface. Unlike systemic VDR inhibition, LNPs loaded with the VDR inhibitor MetC7 retain strong pancreatic targeting ability. This phenomenon indicates that VDR-dependent targeting depends on the amino acid loading on the LNPs surface, rather than on free amines in circulation, and that the resulting VDR-rich corona can also recognize VDR highly expressed in pancreatic β-cells, thereby achieving precise tissue enrichment.

C-CholF3 LNPs can carry all kinds of nucleic acid types, such as mRNA, plasmid DNA, and circular RNA, and have been used to achieve beta-cell-specific gene editing in transgenic mice. This system can also be applied to protein-substitution therapy and CRISPR-based gene editing, and has shown good prospects for managing type 1 diabetes and pancreatic cancer [82,111].

The mechanism from lipid composition to organ tropism can be summarized as a cascade of four steps. LNPs design alters corona composition, which in turn affects receptor binding and ultimately changes organ targeting. This cascade is conserved across different organs but is executed by distinct molecular players. For liver targeting, LNPs rely on ApoE binding to LDLR on hepatocytes, a mechanism validated in LDLR knockout mouse models [24,96]. Lung-targeting platforms center on the Vtn and αvβ_3_ integrin axis on pulmonary endothelial cells. This mechanism is supported by evidence from Vtn depletion, RGD blockade, and proteomic enrichment analyses [94,98,112]. Spleen targeting can be achieved either through β_2_-GPI and CD169-positive macrophage recognition, such as via SORT or 18 PA, or by reducing ApoE adsorption to avoid liver clearance [98]. For lymph node targeting, LNPs adsorb albumin, which binds to gp60 on lymphatic endothelium and triggers transcytosis. This process improves drug retention in the lymph nodes [113], whereas pancreatic targeting exploits VDR recruitment to elicit recognition by pancreatic β-cells, achieving > 99% selectivity [95]. These comparisons reveal a unifying principle: corona composition serves as a more direct predictor of organ tropism than lipid structure alone, and the same corona proteins (e.g., Vtn) can be recruited by structurally diverse LNPs.

##### 3.3.1.3 Dual regulation of protein corona by physicochemical properties of LNP: determining its composition and mediating organ orientation

The physicochemical properties of LNPs, including pKa values, particle size and surface charge, govern the formation and composition of the protein corona, thereby regulating the biological interactions and targeting capabilities of LNPs. Elucidating the relevant structure-activity relationships is crucial for advancing the design of drug delivery systems.

##### 3.3.1.4 pKa

**Fig. 10:**
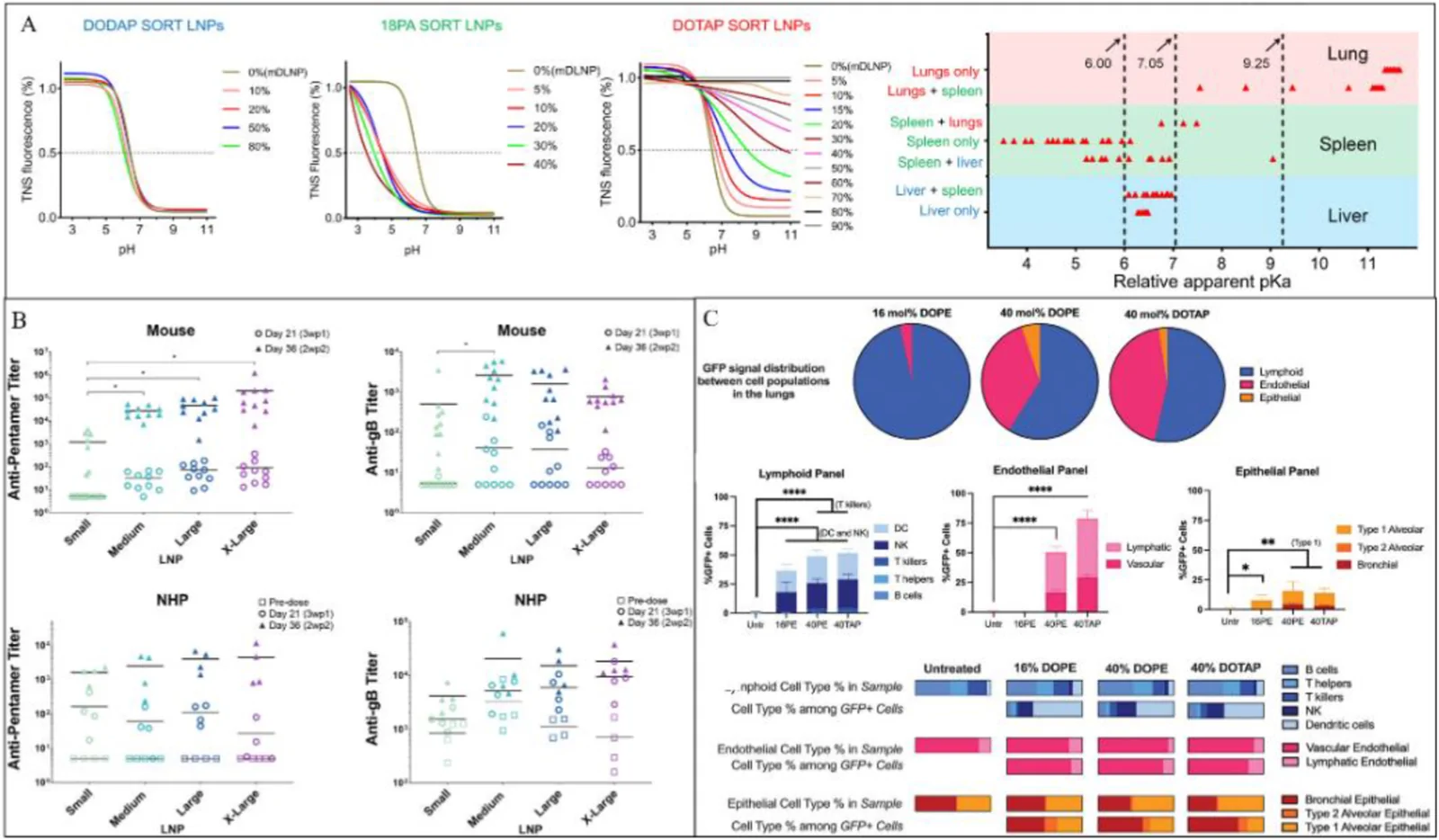
pKa Modulation, Immunogenicity, and Lung Cell Type-Specific Delivery of SORT LNP. A) Representative TNS detection curves used to determine the apparent pKa of SORT LNPs, where the apparent pKa is defined as the point at which TNS fluorescence reaches 50%; and tissue-specific indices are assigned to the LNPs based on tissues in which functional luciferase mRNA is detected, adapted from Ref. [116] with permission from PNAS. Copyright 2021; B) Anti-pentamer and anti-gB IgG titers were measured pre-dose, 3 weeks post-prime, and 2 weeks post-boost following immunization with 64, 81, 108, or 146 nm LNPs encapsulating CMV mRNA. Data were analyzed by Tukey's test (***p < 0.001, reproduced from Ref. [118] with permission from Elsevier. Copyright 2021; C) Total GFP expression in lung cells; GFP expression in lymphoid, endothelial, and epithelial subsets (Untr, untreated; DC, dendritic cells; NK, natural killer cells); and Proportion of total cells versus GFP^+^ cells within the lung, reproduced from Ref. [119] with permission under CC-BY. Copyright 2024.

pKa is the pH at which half of the ionizable lipid species are positively charged. The above parameters are needed to regulate the function of LNPs; they determine both the interfacial charge characteristics in a biological environment and the protonation process within the lipid bilayer [114]. Jayaraman et al. [115] found that the pKa of LNPs significantly influences their siRNA delivery efficiency. Since then, this parameter has become a key reference for designing LNP delivery systems targeting the liver and other tissues such as the lung. In the SORT strategy, adding a fifth charged lipid (e.g., DOTAP) elevates the apparent pKa of the complete LNP formulation and redirects organ targeting: Using the TNS (6-(p-toluidino)-2-naphthalenesulfonic acid) fluorescence assay, apparent pKa values of 6-7, 4-6, and >9 correspond to liver, spleen, and lung targeting, respectively (Fig. 10A) [116], TNS is a lipophilic anionic fluorescent probe. Its fluorescence is quenched in aqueous solution but becomes strongly enhanced upon incorporation into lipid bilayers. As the pH decreases, ionizable lipids become protonated and acquire a positive charge, which attracts the negatively charged TNS into the LNP membrane via electrostatic interactions, producing a pH-dependent fluorescence increase. The apparent pKa is defined as the pH at which the fluorescence intensity reaches half of its maximum value. Unlike this formulation-level apparent pKa, the intrinsic pKa of ionizable lipids typically remains within 6.2-7.0, as exemplified by Yan Li's liver-targeted (∼6.34) and lung-targeted (∼7.04) LNPs [117].

##### 3.3.1.5 Particle size

The size and structure of LNP particles influence targeting by regulating the composition of the protein corona. Larger LNPs (∼100 nm) bind more strongly to ApoE than smaller LNPs (∼50 nm), which increases their affinity for the liver [120]. For DOPC-based LNPs, 160 nm particles accumulate preferentially in pancreatic islets, whereas smaller particles are primarily distributed in exocrine pancreatic tissue [121]. Luis A. Brito [118] was the first to observe a correlation between particle size and immunogenicity in mice, across various formulations with different compositions. To investigate this issue further, he designed a series of experiments that adjusted the LNP particle size while keeping the lipid composition constant, and then characterized the resulting particles' physicochemical properties and immunogenicity. Although smaller LNPs showed significantly lower immunogenicity in mice, all tested sizes still elicited a strong immune response in non-human primates (NHPs) (Fig. 10B).

##### 3.3.1.6 Zeta potential

Javier Gimenez-Warren et al. [122] demonstrated that the surface charge of LNPs, which is modulated by degradable, ionizable lipids containing β-propionate acid bonds, regulates mRNA delivery to specific organs: positive charge promotes expression in the lungs, whereas negative charge directs it to the spleen. This charge-dependent tropism has been mechanistically linked to differences in the composition of the protein corona — a concept that finds an analogue in studies of SORT LNPs [123]. Proteomic analysis confirmed that lung-targeting LNPs selectively recruit Vtn and prothrombin, consistent with established lung-targeting paradigms [124,125].

##### 3.3.1.7 Modifications are made on the traditional four-component formulation: altering the composition of the protein corona to affect targeting properties

Javier Gimenez-Warren and colleagues [122] systematically investigated the structure-activity relationships of naturally occurring ionizable lipids containing β-propionic acid esters, thereby identifying two major molecular carriers for targeted mRNA delivery to specific organs. Regarding the hydrophobic backbone structure, the linear lipid species promoted initial accumulation in lung cells, whereas the same branched species showed directed accumulation in the mammary gland—an effect associated with the high zeta potential of the weakly charged surface. On the other hand, the chemistry of the front group proved to be the controlling independent variable. When the backbone structure was unchanged, the imidazole-containing primary group (which, at physiological pH, slightly loses a proton to become weakly charged) was associated with the cell-directed distribution. On the other hand, the main group derived from a basic cyclic amine (which is always highly protonated to provide a positive zeta potential) directed the particles to be taken up by the lungs. Complementing these findings, Kathryn A. Whitehead [119] proved that branched-tail ionizable lipids enable potent pulmonary mRNA delivery, with cellular resolution governed by helper lipids composition. Notably, incorporation of the neutral inclusive helper lipids DOPE at 16 mol% achieved highly selective transfection of natural killer cells and dendritic cells within the lung microenvironment. The incorporation of the cationic lipid DOTAP enhances delivery to the lungs but reduces cellular selectivity, allowing equal permeation through both endothelial and lymphoid cells. Notably, DOPE-based formulations demonstrated superior biocompatibility over DOTAP-containing counterparts (see Section 4.2 for detailed safety and immunogenicity discussions). Taken together, these data reveal that branched-tail LNPs possess an intrinsic capacity for immune cell-selective pulmonary delivery, operating independently of cationic helper lipids (Fig. 10C).

Daniel J. Siegwart [94] demonstrated that within the cationic lipid category, subtle headgroup modifications exert profound effects on LNP functionality via modulation of the protein corona. Specifically, replacing DOTAP with 18:1 RI reshaped the protein corona and selectively enriched for Vtn, clusterin, albumin, and prothrombin. This Vtn-rich surface layer was mechanistically linked to an increase in the transfection efficiency of pulmonary mRNA, and it has been identified that Vtn functions as a homing signal for the lung [126,127]. These findings corroborate earlier studies on Vtn-corona-mediated lung targeting of SORT LNPs [94,128].

The chemical durability of the linkage in ionizable lipids can serve as a molecular switch to alter the lipophilicity of LNPs, and thus a new design path for tissue-specific delivery has been proposed. When these nanoparticles incorporate labile ester bonds, the bonds are gradually hydrolyzed in circulation, and then different serum proteins attach to the surface of the particles. A lung-targeting protein corona rich in apolipoprotein A4, complement C3, and fibrinogen has formed around the LNPs to help them remain in the pulmonary capillaries and be efficiently phagocytosed by endothelial cells and local immune cells [62]. Deliberately introduce ester bonds that are rapidly cleaved in the acidic endosome to ensure highly specific delivery of mRNA to the lung. The above engineering method has achieved good results and can now shift the aim of biodistribution from the liver to effective, site-specific delivery of nucleic acids in the lungs.

H Lee et al. [129] proposed that rational design of ionizable lipids can control three properties of LNPs: pKa, component biophysical characteristics, and corona formation. Modifying the steric hindrance of the head group, adding ester bonds, and adjusting the length and symmetry of the alkyl chain are used. These modifications enhance ApoE binding, form a protein corona that guides particles to the liver, and at the liver, these coatings are recognized by surface receptors on hepatocytes and other liver cells, such as cholangiocytes, via the sinusoidal endothelium.

Yan Li [117] designed bivalent polymeric ZP-LNPs for organ-selective siRNA delivery by replacing the conventional DMG-PEG lipid with a DMG-PCB lipid. Fine-tune the degree of polymerization, lipid ratio, and lipid-to-siRNA mass proportion to build a 90-formulation library and identify B4-3@siRNA and B5-1@siRNA. Both exceeded 85% siRNA encapsulation and targeted the liver and lungs, respectively. Several protein coronas have been identified by mechanistic analysis that are responsible for this organ tropism. Liver-targeting B4-3@siRNA binds fibrinogen α-chain to increase Kupffer cell uptake and hepatic accumulation [130]. Lung-homing B5-1@siRNA, on the other hand, forms a fibronectin-rich corona that binds to integrin αvβ_1_ on pulmonary endothelial cells and alveolar macrophages and aligns with the known lung-targeting pathway [131].

Qiang Cheng et al. [132] successfully designed and synthesized more than 120 peptidizable lipids (PILs) with diverse structures using modular solid-phase peptide synthesis (SPSS). These constructs are assembled from natural amino acids (or functional building blocks) and Fmoc-protected, ionizable alkylated amino acids (AIFA), thereby expanding the chemical space for programmable organ targeting. Systematic studies of the structure-activity relationship (SAR) and structure-selectivity relationship (SSR) of PILs have shown that the alkyl chain length, chain type, number of AIFA, side chain length, and terminal modifications all affect mRNA delivery efficiency and organ selectivity. By synthesizing a panel of PILOT LNPs functionalized with amino acids of different physicochemical properties, the authors found that positively charged amino acid-modified PILs greatly improved mRNA expression in both hepatic and splenic tissues. In comparison, tyrosine-functionalized PILOT LNPs achieved specifically elevated expression in the spleen alone. Furthermore, the introduction of a lysine-tyrosine dipeptide unit directed the nanoparticles toward the thymus, leading to substantially strengthened mRNA expression in the thymus and simultaneously decreased expression in other off-target organs. Separately, functionalization with the bone-targeting bisphosphonate alendronate sodium (Ale) drove substantial mRNA accumulation in both femur and tibia. These results unequivocally demonstrate that amino acid modifications can endow the PILOT platform with broad tissue-targeting potential. Follow-up mechanistic investigations illustrated that the tissue tropism mediated by PILOT LNPs does not rely on classical active targeting pathways based on specific ligand–receptor recognition. Rather, this organ-selective distribution is realized by modulating the profile of proteins adsorbed onto the nanoparticle surface during in vivo circulation, namely the protein corona. First, the researchers incubated PILOT LNPs—each exhibiting distinct tissue tropism—with mouse plasma to recapitulate protein corona formation under physiologically relevant conditions. Following SDS-PAGE analysis of protein corona components isolated from the nanoparticle surface, formulation-specific protein bands were observed. This result suggests that the structure and makeup of the adsorbed protein shell are closely associated with the LNP formulation design. This indicates that the proteins adsorbed differed in both type and/or quantity. Subsequently, the researchers analyzed potential reasons for the differing Protein corona compositions: they discovered that introducing additional charges at the N- or C-terminus (positively charged amino groups or negatively charged carboxyl groups) significantly enhanced spleen targeting, indicating surface charge is a key factor influencing protein adsorption and organ selectivity. Furthermore, pKa values, tail chain structures, and linker bonds may also exert influence. Based on this, the researchers made an important discovery: in vitro experiments showed that cellular uptake largely depends on the positive surface charge of the LNPs. However, in vivo, many highly effective PLs have an almost neutral or negative surface charge. This discovery suggests that, in vivo, the protein corona masks the intrinsic charge of LNPs and creates a new interfacial feature that governs subsequent cellular interactions. Although it is not yet possible to rationally design amino acid modifications to confer tissue-specific affinity to the corona, these findings establish the protein corona as a key link between LNP composition and selective tissue delivery. They also provide deeper insight for future research on LNP-mediated extrahepatic targeting [132].

Michael J. Mitchell [112] used parallel synthesis to create a library of 252 ionizable lipids with siloxane modifications and thus a diverse set of siloxane-doped LNPs. The addition of these siloxane groups expands the effective radius of the head group, increases the deformability of the bilayer, and thus enhances both cell entry and endosomal escape for tissue-selective mRNA delivery. Among the candidates, the liver-tropic Si4-C14b formulation showed an apparent pKa of 6–7 and selectively bound ApoE to promote hepatocyte uptake via the LDLR pathway.

Si5-N14 LNPs also had good lung-targeting capabilities. In a model of influenza-induced pulmonary injury, Si5-N14 LNPs loaded with FGF-2 mRNA bound to Vtn-integrin interactions to anchor damaged pulmonary endothelium, promote vascular repair, and restore oxygen saturation and alveolar architecture. In a lung cancer model, Si5-N14 LNPs were used to edit the VEGFR2 gene in pulmonary endothelial cells to achieve high-selectivity anti-angiogenic effects in the lungs. Mechanistic interrogation shows that Vtn is highly concentrated in the protein corona and ApoE adsorption is reduced; thus, LNP tropism has been shifted away from the liver [96,133]. Based on the above observations, it can be concluded that glycoproteins likely serve as the main factors determining the lung-specific targeting ability of LNPs. Unfortunately, the essential proteins that regulate splenic homing of LNPs have not yet been identified.

Additionally, Pieter R. Cullis [134] engineered a structurally unique liposomal formulation [134] that extends the circulation time by altering the biomolecular corona. This design reduces the binding of immunostimulatory proteins; thus, decreased immunoglobulin deposition inhibits Fc-receptor-mediated phagocytosis and subsequent clearance by the liver [135,136], while reduced fibrinogen recruitment mitigates coagulation-mediated clearance through the MPS [137,138]. At the same time, elevated enrichment of apolipoproteins A1 and E creates a high-density-lipoprotein-like surface coating, which encourages LDLR-facilitated internalization and supports dispersal to tissues beyond the hepatic compartment [21,139,140].

Gong Cheng [113] engineered LNPs to recruit albumin, facilitating lymphatic drainage to lymph nodes while limiting systemic and hepatic accumulation. An albumin-binder (AB) ionized lipid library was generated, with AB-lipids replacing PEG-lipids to prepare albumin-recruiting AB-LNPs; Evans blue-modified lipid (EB-lipid)-based EB-LNP showed optimal performance. Its mechanism involves: (1) EB-lipid-mediated albumin adsorption onto the LNP surface, establishing a defined protein corona; (2) After intramuscular injection, EB-LNP surface recruit albumin to form a protein corona, which interacts with lymphatic endothelium to generate an osmotic pressure gradient that drives LNP transport through lymphatic vessels to draining lymph nodes, while reducing its chances of entering capillaries. Therefore, the systemic circulation and subsequent liver deposition are significantly restricted [141]; (3) Through dendritic cells (DCs) present in the lymph nodes, the use of albumin receptors (such as gp60) facilitates efficient endocytosis of EB-LNP, leading to a strong immune response.

Given the multitude of factors governing alterations in LNP-targeted behavior, we have classified LNPs according to their target organs and noted the changes in their formulation, protein corona and physicochemical properties, with a view to providing readers with a reference when investigating the reasons why LNPs target different organs (Table 1).

**Table 1:** Optimized landscape of LNP-Mediated extrahepatic targeting: From chemical composition to biological identity.

Target organ	Standardized Formulation (mol%)	Targeting Strategy & Molecular Mechanism	Biological Identity (Key Corona Proteins) & Physicochemical Traits	Ref.
Lung	mRNA: IAJD34 (weight ratio) = 1:40	IAJD34: Achieving targeted modification through the screening of ionizable lipids	**Physicochemical Traits:** Size, 95–123; nm; pKa, 6.74.	[142]
	306-N16B:50% Cholesterol:38.5% DOPC:10% DMG-PEG:1%	306-N16B: Achieving targeted modification through the screening of ionizable lipids	**Protein coronas:** Serum albumin, Fibrinogen β chain, Fibrinogen γ chain **Physicochemical Traits:** Size, 80–120; nm, Potential, −5 mV.	[62]
	5A2-SC8:11.9% Cholesterol:23.8% DOPE:11.9% DMG-PEG:2.4% DOTAP:50%	DOTAP: By incorporating a fifth component—lipids—into the existing four-component formulation, its targeting properties are altered.	**Protein coronas:** Vtn, Albumin, Fibronectin.	[98]
	IA3T2C7:50% Cholesterol:38.5% DOPE:10% DMG-PEG:1.5%	A3T2C7: Achieving targeted modification through the screening of ionizable lipids	**Protein coronas:** Vtn, Prothrombin, Fibrinogen beta chain; **Physicochemical Traits:** Size, 70–110 nm, Potential, 0 -+3.5 mV.	[122]
	5A2-SC8:23.8% Cholesterol:47.6% DOPE:23.8% DMG-PEG:4.8% Quaternary ammonium cationic lipids(TAP or RI):50%	18:1TAP By incorporating a fifth component—lipids—into the existing four-component formulation, its targeting properties are altered.	**Protein coronas:** Vtn, Prothrombin, Clusterin; **Physicochemical Traits:** Size, 110–140 nm; Potential, 0∼+7 mV.	[94]
		18:1RI: By incorporating a fifth component—lipids—into the existing four-component formulation, its targeting properties are altered.		
	Siloxane-lipidoid:35% Cholesterol:46.5% DOPE:16% DMG-PEG:2.5%	Siloxane-lipidoid: Achieving targeted modification through the screening of ionizable lipids	**Protein coronas:** Vtn, Serum Albumin, Apolipoprotein; **Physicochemical Traits:** Size, 86 nm; Potential, +2.6 mV.	[112]
	A2-3O14:50% Cholesterol:38.5% DOPE:10% DMG-PEG:1.5%	A2-3O14: Achieving targeted modification through the screening of ionizable lipids	**Protein coronas:** Vtn, Fibrinogen, Fibronectin; **Physicochemical Traits:** Size, 100–130 nm; Potential, +2 ∼ +5 mV	[143]
	Ionizable lipids:50% Cholesterol:38.5% Helper lipids:10% DSPE-PEG:0.75% Peptide-modified lipid DSPE-PEG-6R (RRRRRR/lung-targeting): 6% R: Arginine	DSPE-PEG-Peptide: Modifying target peptides on LNPs, wherein the peptide segments are exposed on the LNP surf-ace, confers unique properties. Different peptide sequences can selectively adsorb varying types and quantities of plasma proteins, forming distinct protein caps. This process forms the basis of their targeting capability.	**Protein coronas:** Vtn, Secreted phosphoprotein 2, Immunoglobulin-like proteins; **Physicochemical Traits:** Size, 80–100 nm; Potential,+20 ∼ +50 mV.	[14]
	Ionizable lipid:50% Helper lipids:10% Cholesterol:38.5% DMG-PEG:1.5% LNP surface-modified CD47 peptides	1. Surface modification with CD47 peptide reduces uptake by macrophages and liver tissues in vivo; 2. Conjugating CD47 peptide with a targeting antibody confers specific targeting capability In contrast to corona-mediated endogenous targeting (SORT/POST), this strategy relies on direct peptide–receptor recognition rather than on protein corona reprogramming.	**Physicochemical Traits:** Unmodified LNP Size:75.7 ± 0.3 nm, Potential:−6.88 ± 0.52 mV; CD47-LNP Size: 76.8 ± 0.4 nm Potential: +2.72 ± 1.82 mV	[87]
	sulfonium lipid(DHSEH or DOSEH):50% Cholesterol:10% DMG-PEG:1.5% DOPC:10%	sulfonium lipid: Achieving targeted modification through the screening of ionizable lipids	**Protein coronas:** Fibrinogen alpha chain, Serine protease inhibitor A3K, Apolipoprotein A-1; **Physicochemical Traits:** Size (DHSEH): 244 nm; Size (DOSEH): 188 nm Potential (DHSEH): +42.5 mV Potential (DOSEH): +46.4 mV	[144]
Spleen	A3B7C2:50% Cholesterol:38.5% DSPC:10% DMG-PEG:1%	A3B7C2: Achieving targeted modification through the screening of ionizable lipids	**Protein coronas:** IMIL-A3B7C2 surface lacks ApoE (reduced hepatic uptake) **Physicochemical Traits:** Size, 105.5 ± 1.3 nm; Potential: −3.3 ± 0.5 mV	[145]
	5A2-SC8:16.7%; Cholesterol:33.3%; DOPE:16.7%; DMG-PEG:3.3%; 18 PA:30%	18 PA: By incorporating a fifth component—lipids—into the existing four-component formulation, its targeting properties are altered.	**Protein coronas:** β_2-_GPI, Albumin, Fibrinogen β chain	[98]
	CL15F 6-4:50% Cholesterol:38.5% DSPC:10% DMG-PEG:1.5%	CL15F 6-4: Achieving targeted modification through the screening of ionizable lipids	**Protein coronas:** Short-tailed CL15F 6-4 reduces ApoE adsorption thereby preventing hepatic uptake; **Physicochemical Traits:** Size, 90 nm; Potential, negative potential.	[71]
	A2T2C9:50% Cholesterol:38.5% DOPE:10% DMG-PEG2000:1.5%	A2T2C9: Achieving targeted modification through the screening of ionizable lipids	**Protein coronas:** Actin, Myosin, Immunoglobulin lambda-like polypeptide **Physicochemical Traits:** Size, 70–110 nm; Potential, –9 ∼ –19 mV.	[122]
	Ionizable lipid:50% Cholesterol:38.5% Helper lipids:10% DSPE-PEG:0.75% Peptide-modified lipid DSPE-PEG-6D (DDDDDD/spleen-targeting): 6% D (aspartic acid)	DSPE-PEG-Peptide: Modifying target peptides on LNPs, wherein the peptide segments are exposed on the LNP surface, confers unique properties. Different peptide sequences can selectively adsorb varying types and quantities of plasma proteins, forming distinct protein caps. This process forms the basis of their targeting capability.	**Protein coronas:** Sparcl1(Secreted protein acidic and cysteine rich-like 1), Clec1b(C-type lectin domain family 1 member B), Fam114a2(Family with sequence similarity 114 member A2) **Physicochemical Traits:** Size:90-110 nm; Potential, –20 ∼ –30 mV.	[14]
	nor-MC3: 20% ESM:40% Cholesterol:40% PEG-DMG:1.5%	Incorporation of ESM forms a bilayer lipid structure. A high proportion of bilayer lipids enables LNP to adsorb fewer proteins in plasma, thereby prolonging its half-life in vivo.	**Protein coronas:** Albumin, Apolipoproteins, Immunoglobulins (IgG/IgM); **Physicochemical Traits:** Size, 40–60 nm; Potential, Approaching neutral.	[134]
Kidney	DOPE:9.5% CHEMS:2% DSPE-PEG:1% R8 (octa-poly-L-arginine):5% (relative to total lipids)	R8 (octahydroxyarginine) is a cell-penetrating peptide (CPP) that enhances cellular uptake and drug retention in the glomerulus.	**Physicochemical Traits:** Size, 110 nm; Potential, +6.64 ± 0.83 mV.	[146]
	Soybean Lecithin:15 mg Cholesterol: 3 mg DSPE-PEG2000:1 mg BRNC (Bilirubin Nanocrystals):1.9 mg (Lipid ratio 1:10)		**Physicochemical Traits:** Size, 76.88 ± 0.44 nm; Potential, −21.69 ± 0.64 mV.	[147]
	DOTAP:50% 5A2-SC8:11.9% DOPE:11.9% Cholesterol:23.8% DMG-PEG2K:2.38%	By increasing the DOTAP ratio, the LNP acquires a strong positive charge, thereby adsorbing negatively charged Vtn to form a protein cap. Acting as an endogenous ligand, Vtn mediates the binding of LNP to αvβ_3_ integrin, enabling receptor-mediated endocytosis and facilitating LNP accumulation in the kidneys.	**Physicochemical Traits:** Size, 100 nm; Potential, +40 mV.	[107]
Brain	A2-B13:39% DOPC:30% Cholesterol:30% DMG-PEG:1%	A2-B13: A2-B13 is a tetrahydroisoquinoline derivative of berberine with DRD3 antagonistic activity. By binding to the dopamine D3 receptor (DRD3) in the brain, A2-B13 mediates transport across the blood-brain barrier (BBB).	**Physicochemical Traits:** Size(BE-LNP in solution), 70 nm; Size(BE@siRNA complex), 90 nm; Potential (BE@siRNA complex): slightly negative or neutral.	[148]
	Ionizable lipids (furan/tetrahydrofuran derivatives): 22% Cholesterol:44.1% DOPE:33.1% DMG-PEG: 0.8%	F10T5/F11T6: Achieving targeted modification through the screening of ionizable lipids.	**Physicochemical Traits:** Size (F10T5), 170 nm; Size (F11T6), 154 nm; Potential (F10T5), negative potential; Potential (F11T6), negative potential.	[149]
	Ionizable lipid:50% DSPC:10% Cholesterol:38.5% DMG-PEG:1.5% DSPE-PEG-maleimide:10% Targeted peptides (4 types)	1. DSPE-PEG-mal- provides a linker site for the peptide segments, enabling their successful loading onto LNPs. 2. Four distinct peptide segments are surface-modified onto LNPs to confer targeting capabilities.	**Physicochemical Traits:** Size (RVG29-LNP), 95 ± 4 nm; Sizem (ApoE-LNP), 92 ± 3 nm; Size (AP2-LNP), 105 ± 6 nm; Size (T7-LNP), 89 ± 2 nm; Potential (RVG29-LNP), –4.0 ± 0.7 mV; Potential (ApoE-LNP), +1.5 ± 0.9 mV; Potential (AP2-LNP), –2.8 ± 0.8 mV; Potential (T7-LNP), –3.5 ± 0.6 mV.	[150]
Heart	Ionizable lipid: 50% DSPC: 10% Cholesterol: 38.5% DMG-PEG: 1.5% Platelet membrane outer coating	The expression of CD47 on the platelet membrane surface inhibits macrophage phagocytosis, reducing hepatic and splenicsequestration. Platelet membranes retain a degree of fusion capacity, enabling localized fusion or endocytosis with phosphatidylserine (PS) and integrins on cardiomyocyte surfaces, thereby mediating LNP entry into cardiomyocytes.	**Physicochemical Traits:** Size (Uncoated), 100 nm; Potential (Uncoated), negative potential; Size (coated), 110 nm; Potential (coated), negative potential.	[151]
	ALC-0315: 47.4% Cholesterol: 40.8% DSPC: 10.3% DMG-PEG: 0.5% ATTO700-DPPE Fluorescent-Labelled Lipid:1%	The cardiac enrichment relies on the administration route rather than LNP design. Direct intracoronary injection makes the heart a first-pass organ, reducing dilution in the liver and spleen, but does not achieve systemic programmable targeting via “biological identity” engineering.	**Physicochemical Traits:** Size, 120 nm; Potential, −5 to −10 mV.	[152]

#### 3.3.2 Functional expansion of protein coronas: endogenous targeting (peptide-primed POST included) and immune regulation

Targeting strategies are classified according to the core mediators of organ uptake, rather than by the presence or absence of surface modifications. Within this framework: (1) intrinsic corona-mediated targeting (SORT, ENDO) does not require surface conjugation; (2) peptide-induced endogenous targeting (POST) utilizes surface-tethered short peptides that do not directly bind to receptors but rather remodel the protein corona to recruit endogenous organ-homing proteins; and (3) classical covalent active targeting (e.g., CD47 peptide, RVG peptide LNPs) relies on surface-conjugated ligands that directly bind to cell surface receptors to drive uptake. Thus, the difference between POST and active targeting is at the level of function: POST mediates targeting by the protein corona and is therefore considered endogenous targeting, while active targeting is achieved through ligand-receptor interactions.

Protein coronas not only serve as biomarkers but also regulate the targeting of LNPs. They also possess targeting potential and participate in immune regulation. This dual functionality provides a valuable strategy for improving drug delivery precision. With its ability to deliver LNPs to specific locations and modulate the immune response, the protein corona has opened new possibilities in the design of next-generation targeted therapies [153,154].

##### 3.3.2.1 Active targeting formulations with specific components

Giulio Caracciolo et al. [155] reported that DOTAP/DNA lipoplexes, in particular, contain Vtn and albumin in human blood plasma. Vtn helps bind to RGD αvβ_3_ integrin—which is highly expressed in tumors and in the formation of new blood vessels—indicating that the corona formulation facilitates passive delivery of the corona to tumors. To confirm the effect of the protein corona on the targeting of specific sites, the study used flow cytometry on αvβ_3_-positive MDA-MB-435S cells and αvβ_3_ compared the uptake of lipid DOTAP/DNA complexes and protein DOTAP/DNA complexes in αvβ_3_-negative HEK293 cells: In MDA-MB-435S cells expressing high levels of αvβ_3_, protein corona increased lipoplex uptake by approximately fivefold; On the other hand, In HEK293 cells, which had a lower level of αvβ_3_ expression, a slight increase (approximately twofold) was observed, indicating that the fibronectin–αvβ_3_ pathway is the primary mechanism of uptake. Fluorescence microscopy confirmed this interaction, revealing colocalization between several regions of the lipoplex and αvβ_3_-tagged GFP; This co-localization was significantly reduced after the addition of a soluble RGD peptide, confirming that Vtn is RGD-dependent, mediates direct integrin-dependent endocytosis, and this recognition can be competitively inhibited [[155], [156], [157]]. Overall, these findings show that specific parts of the protein corona can cause the LNPs to be actively targeted, thereby increasing the chances of achieving precise targeting of the organs. This is possible thanks to pre-coating strategies where the LNPs are covered with selected proteins prior to administration.

##### 3.3.2.2 Bidirectional regulation of immunogenicity by the protein corona

In addition to targeting cells, protein corona also plays an important role in regulating immune responses. Ashish Kulkarni et al. [158], investigated the immunogenicity of LNP2, a synthetic lipid-binding compound (not a complete LNP formulation). In serum-free assays, LNP2 strongly activated the NLRP3 inflammasome, as evidenced by increased IL-1β secretion and ASC speckle formation. Under pharmacological inhibition, this activity decreased by 70%, confirming that LNP2 has intrinsic immune-stimulatory activity even in the absence of a protein corona. It is important to note that these experiments were conducted under serum-free conditions, meaning no protein corona could form on the surface of the compound. Therefore, the observed NLRP3 activation reflects the intrinsic immune-stimulatory properties of the lipid molecule rather than coating-mediated effects. However, under physiological conditions (e.g., in the presence of serum), pre-existing protein capsids can adsorb onto LNP2 or related LNP formulations, altering their surface properties and modulating inflammasome activation, This can occur by protecting the underlying lipid moieties or by attracting complement-inhibitory proteins [159]. Thus, while LNP2 exemplifies how lipid-based carriers can intrinsically trigger immune pathways, the protein corona often acts as a suppressive buffer in vivo, complicating direct extrapolation from serum-free data. A thorough evaluation of LNP immunogenicity should therefore compare both serum-free and serum-containing conditions to disentangle intrinsic effects from corona-mediated regulation. Therefore, these observations collectively indicate that the protein corona both affects target distribution and modulates immune responses, highlighting the need for a comprehensive assessment during the rational design of LNP system.

### 3.4 A comparison of extrahepatic targeting platforms and their limitations

The SORT, POST, ENDO and other platforms discussed in this article have their own advantages and disadvantages, but all have unresolved key issues. We hope to provide some reference directions for our researchers in the process of universal research and clinical translation of these technology platforms by raising these questions.

SORT technology is easy to operate and only requires the addition of a fifth charged lipid, which can deliver mRNA and siRNA. But organ selectivity is not absolute, and the liver still has 10-30% off target accumulation. Changing surface charge can lead to non-specific protein adsorption and toxicity, such as DOTAP causing cytokine release. The optimal ratio needs to be optimized separately for each LNP. The impact of SORT lipids on long-term toxicity and repeated administration is still unclear. There is no evidence of the effectiveness of non-invasive administration of SORT for difficult to target organs such as the brain and pancreas.

The POST technology regulates the protein corona through peptide sequences, with higher accuracy and compatibility with different ionizable lipids and helper lipids. Changing a single amino acid can switch organ orientation, with strong programmability. The disadvantage is that peptide conjugation requires chemical synthesis and linkers, which is more complex and costly than SORT. At present, there are few successful sequences (such as 6R, 6D), and it is not yet possible to predict protein corona composition from peptide sequences. There is a lack of data on whether POST can further target specific cell types from organs, as well as the immunogenicity and in vivo stability of peptides.

The ENDO platform utilizes vitamin D3 to recruit VDR from plasma, achieving a targeting efficiency of>99% in mouse pancreas. It is suitable for various nucleic acids and has low toxicity. But this mechanism has not been independently validated yet. Dependence on vitamin D3 may limit its use in other organs. Efficient targeting has only been reported in pancreatic islets, and it is uncertain whether it is applicable to other tissues.

In addition, the types of RNA, animal models, doses, and detection indicators used in different studies are different, making it impossible to directly compare different platforms. The same lung targeting formula may be effective for mRNA, but may not be effective for siRNA; The peptide sequence that is effective for SM-102 may not be useful if replaced with other lipids. The toxicity and immunogenicity data mostly come from single dose administration, and anti-PEG antibodies and accelerated blood clearance (ABC) phenomenon become prominent during repeated administration. Large scale production and inter batch reproducibility are rarely discussed, which are practical obstacles on the road to clinical translation. From a mechanistic perspective, these platforms also differ fundamentally in how they rewrite the LNP's biological identity. SORT works by first adding a charge; then, the altered surface potential selectively attracts certain corona proteins (such as Vtn in the lung and β_2_-GPI in the spleen) through electrostatic and hydrophobic interactions. POST is a “sequence-encoded" type that controls protein adsorption to alter the corona, thus regulating the corona-receptor axis more precisely. ENDO is a “molecular chaperone" that uses a small molecule (vitamin D) to recruit a specific endogenous protein (VDR) to the corona and achieves exceptional selectivity for a single organ (pancreas). A small table showing the above technologies is provided for easy reference (Table 2).

**Table 2:** Comparison of SORT, POST, and ENDO platforms.

Platform	Key Mechanism	Advantages	Major Limitations	Validated Organs
SORT	Add 5th charged lipid → alter protein corona	1. Simple, no conjugation	1. 10-30% liver off-target 2. Charge toxicity (DOTAP) 3. No brain/pancreas data	Lung, spleen, liver, kidney
POST	Peptide-conjugated lipid → selective protein adsorption	1. Programmable (single amino acid) 2. Cross-formulation compatible(Suitable for various lipid formulations)	1. Peptide synthesis needed 2. Few known sequences 3. Immunogenicity unknown	Lung, spleen
ENDO	Vitamin D_3_ → recruits VDR corona → Targeting pancreatic β-cells	1. >99% pancreas targeting 2. Low toxicity	1. Mechanism unvalidated 2. Vitamin D_3_-dependent 3. Only pancreas reported	Pancreas

Although the mechanistic designs of SORT, POST, and ENDO platforms differ considerably, they share a common fundamental limitation: all rely on the assembly of a dynamic protein corona, a process inherently susceptible to variations in plasma composition, fluid dynamic conditions, and inter-individual differences—factors that collectively undermine the predictability of the design. Mechanistically, SORT is constrained by its charge-driven protein recruitment, which lacks molecular specificity and results in broad protein adsorption, with formulation optimization relying largely on empirical screening. POST faces challenges related to peptide instability in circulation and the yet-to-be-elucidated relationship between peptide sequence, corona composition, and organ tropism. ENDO, despite achieving promising pancreatic specificity, is limited by the species-dependent nature of its VDR-recruitment mechanism and its restricted applicability beyond pancreatic tissues. Beyond these platform-specific issues, the poor reproducibility observed across different animal models further stems from substantial inter-species differences in plasma proteomes (e.g., ApoE, Vtn).

## 4 Organ-specific delivery case: beyond the liver

### 4.1 Organ-specific delivery: case studies

This section examines recent advances in extrahepatic LNP delivery across six major organs, with emphasis on the mechanistic roles of the three-pillar framework. Route-dependent organ enrichment via local administration is explicitly noted to distinguish it from systemically programmable targeting.

#### 4.1.1 Targeted delivery therapy for the lung

Respiratory cilia are easy-to-target and essential components for pulmonary nucleic acid therapies of refractory lung diseases, such as cystic fibrosis and lung cancer. However, intrinsic biological barriers in the lung, such as mucociliary clearance and phagocytosis by resident alveolar immune cells, limit both the retention period and gene-transfer efficiency of topically applied LNPs [[160], [161], [162], [163]].

To overcome these constraints, scientists have engineered a range of refined LNP systems designed to home to pulmonary tissue for disease-tailored therapeutic applications. Zhao et al. engineered LNPs that target endothelial cells, named LuLNPs, to deliver VEGFA mRNA. This approach restored pulmonary regenerative function after influenza induced TGFBR2 deficiency [164]. Li et al. [165]. reduced the LNP formulation to three components with no helper lipids, improving delivery efficiency by 35 times. In two rat models of pulmonary arterial hypertension, BMPR2 mRNA loaded LNPs reversed vascular remodeling and delayed disease progression.

**Fig. 11:**
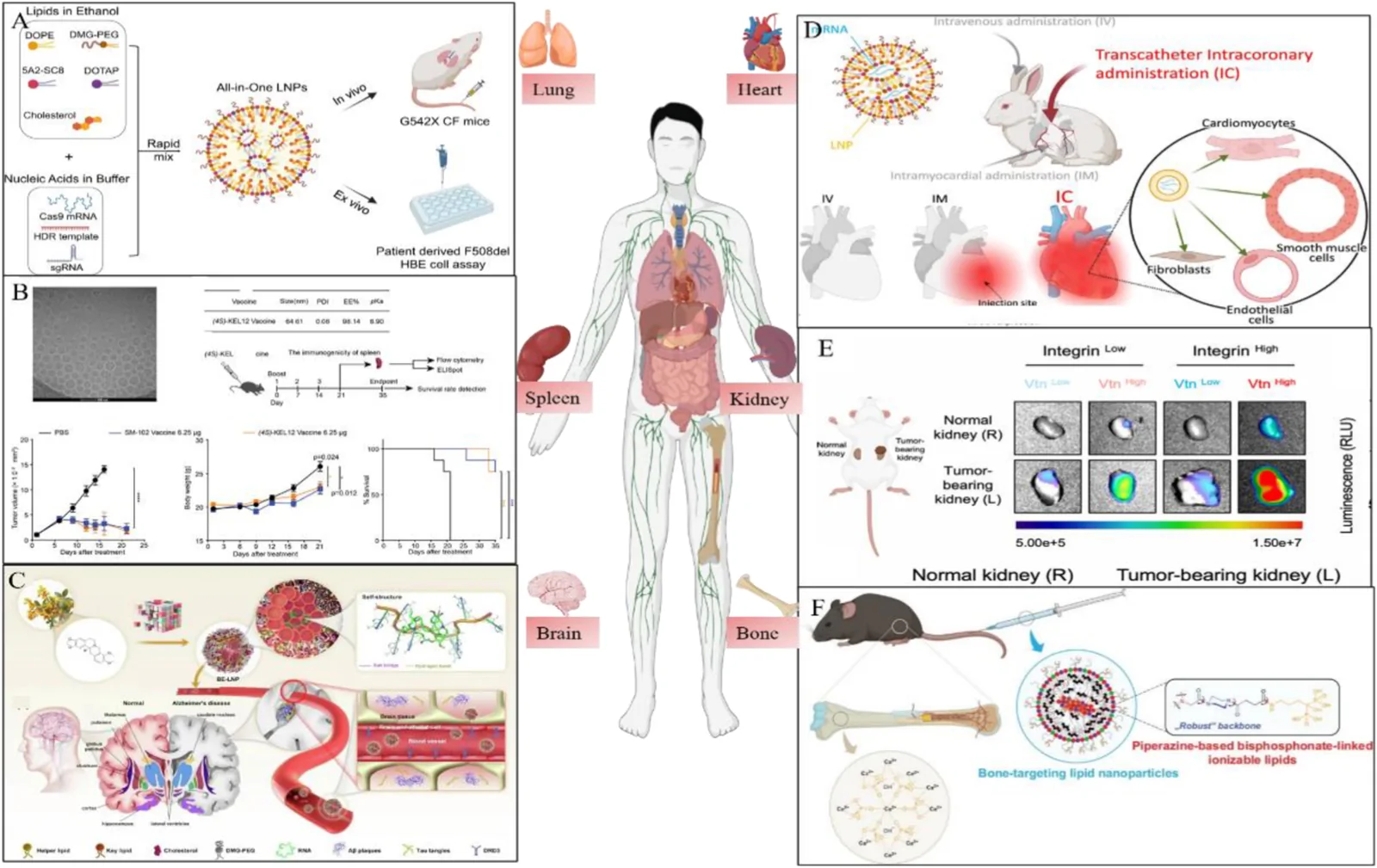
Organ-specific delivery case: Beyond the liver. A) SORT LNPs to deliver CFTR mRNA, reproduced from Ref. [166] with permission under CC-BY 4.0. Copyright 2023; B) Cryo-EM micrographs of the (4S)-KEL12 vaccine, its physical properties, tumor volume (center left), body weight (center right), and animal survival rate (right) in tumor-bearing mice following three weekly injections of the specified dose; comparison of PBS as a negative control with SM-102 and the (4S)-KEL12 vaccine following intramuscular injection of 6.25 μg, adapted from Ref. [170] with permission under CC-BY. Copyright 2024; C) Construction of a three-dimensional combinatorial synthesis library and formation of BE@RNA complexes via berberine derivative-poly(A) affinity; blood–brain barrier penetration mediated by dopamine D3 receptor (D3R) transport for targeted therapy in neurodegenerative disorders, reproduced from Ref. [148] with permission under CC-BY-NC-ND 4.0. Copyright 2025; D) effective intracoronary delivery of Cardiac-Targeted mRNA-LNP via catheter, adapted from Ref. [152] with permission under CC-BY. Copyright 2025; E) In vivo quantification and imaging of luciferase expression in patient-derived ccRCC tumors orthotopically implanted in mouse kidneys; LNPs encapsulating luciferase mRNA were administered intravenously, with ex vivo imaging at 6 h post-injection, adapted from Ref. [107] with permission from American Chemical Society. Copyright 2025; F) Piperazine-based bisphosphonate-linked ionizable lipids for improved mRNA-LNP delivery to bone, reproduced from Ref. [171] with permission under CC-BY. Copyright 2024.

In the treatment of cystic fibrosis, the SORT LNPs optimized by Siegwart's team [166] successfully delivered CFTR mRNA, restoring CFTR protein expression and chloride ion transport function in G542X mutant mice and patient-derived cells with the F508del mutation (Fig. 11A). In the field of pulmonary fibrosis, Cao et al. [167] developed lung-targeted dLNPs carrying siRUNX1. This formulation selectively accumulates in damaged lung tissue, thereby exerting potent anti-fibrotic effects while minimizing systemic off-target toxicity. In the diagnosis and treatment of lung cancer, gadolinium-doped LNPs enable lung-specific mRNA delivery and dual-modality MRI/bioluminescence tumor imaging, thereby improving the sensitivity of early diagnosis for malignant thoracic tumors [168]. Mitchell et al. [169] screened a series of CAD lipid libraries and identified four high-purity lung-targeted LNPs with negligible distribution in the liver and spleen. By delivering Cas9 mRNA/VEGFR2 sgRNA, these LNPs inhibited tumor angiogenesis and prolonged survival in lung tumor models, outperforming conventional MC3/DOTAP formulations.

Although current LNP-mediated lung targeting has yielded encouraging results in preclinical studies of various respiratory diseases, several challenges continue to limit their translational application. Most existing studies have primarily focused on short-term therapeutic effects in rodent models. Their long-term pulmonary biocompatibility, cumulative immune stimulation, and stability under repeated dosing have yet to be fully validated. Furthermore, most lung-targeting strategies rely on passive tissue accumulation and chemotaxis, lacking precise cell-type-specific targeting control. The heterogeneity of pulmonary lesions and interindividual variations in mucosal barrier function further compromise the consistency of drug delivery and the reproducibility of treatment. Furthermore, currently reported LNPs for diagnostic and therapeutic applications involve relatively complex synthesis processes, which limit their large-scale standardized production and subsequent clinical translation.

#### 4.1.2 Targeted delivery therapy for the spleen

The spleen is the largest secondary lymphoid organ in the human body and plays a central role in systemic immune regulation and tumor-specific T-cell activation, making it an ideal target for cancer immunotherapy. By rationally designing spleen-tropic LNPs, it is possible to achieve targeted immune regulation, minimize peripheral toxicity, and enhance antitumor immune responses [76,172]. Wang et al. prepared LNPs doped with saturated fatty acids, thereby achieving spleen-selective mRNA translation and a potent anti-lymphoma immune response [173]. Lv et al. developed LNPs based on degradable ketoester lipids (KEL); in a mouse tumor model, the optimized (4S)-KEL12 mRNA vaccine induced potent cellular immunity, tumor regression, and prolonged survival (Fig. 11B), similarly, compared with conventional MC3 LNPs, hydroxychloroquine-derived ionizable LNPs exhibited superior splenic targeting and anti-metastatic efficacy [170]. To address the issue of immunogenicity associated with repeated dosing, the Wang research group designed low-immunogenicity GM-lipid LNPs. This formulation avoids the generation of anti-PEG/anti-GM antibodies, maintains stable efficacy after multiple injections, and demonstrates superior performance in splenic mRNA delivery and tumor protection compared to conventional PEG-LNPs [174].

mRNA immunotherapies targeting natural killer (NK) cells have demonstrated broad antitumor and antiviral potential, but delivering mRNA to NK cells in the spleen remains challenging. Liu et al. [175] constructed a library of 161 carbonate-ion-containing lipids (CAILs) for the preparation of intravenously administered LNPs. The four-component CAIL-LNPs exhibited significantly reduced liver accumulation and preferential spleen accumulation; the removal of cholesterol further enhanced their splenic targeting, outperforming previously reported spleen-targeting nanocarriers. The optimized CAIL-LNPs achieved a 21% transfection efficiency in Ai9 splenic NK cells, providing a new blueprint for spleen-targeted NK cell-based mRNA therapy.

Nevertheless, current LNP strategies targeting the spleen still rely on empirical lipid screening. The mechanisms that link lipid composition, protein corona modifications, and splenic immune cell selectivity remain poorly understood. Moreover, most studies have focused on acute antitumor immune responses, but the long term effects on immune homeostasis and possible exhaustion caused by splenic LNP stimulation still need systematic evaluation. Recent work has expanded splenic and lymphatic targeting for cancer immunotherapy. One example is a structurally stabilized lipid polymer nanoplatform that targets antigen presenting cells across tissues and enables durable in situ mRNA cancer immunotherapy [176]. Concurrently, LNP-mediated lymph node-targeting delivery of mRNA cancer vaccines has been shown to elicit robust CD8^+^ T cell responses [177], highlighting the potential of lymphoid organ targeting for generating potent antitumor immunity.

#### 4.1.3 Targeted delivery for brain therapy

The BBB maintains homeostasis in the central nervous system (CNS) through highly selective permeability, but it severely impedes the delivery of high-molecular-weight and hydrophilic nucleic acid drugs to the site of brain lesions [[178], [179], [180]]. Current strategies for crossing the blood-brain barrier are primarily divided into two approaches: direct penetration and barrier bypass, which can be applied to therapeutic interventions for neurodegenerative diseases, brain tumors, and stroke [[181], [182], [183], [184]].

Direct carrier optimization is the primary strategy for enhancing blood-brain barrier penetration. Modulating the physicochemical properties of lipids and surface-modifying them with targeting ligands helps promote transcellular endocytosis across the blood-brain barrier. Li et al. found that berberine (BE) not only exhibits good antidiabetic properties [185], but they also developed a BE-ST formulation; this LNP can efficiently deliver siRNA, mRNA, and small molecules across the BBB for the treatment of neurodegenerative diseases and brain tumors (Fig. 11C) [148]. Wang et al. [186] developed neurotransmitter-derived NT-lipids for the systemic delivery of small molecules, genes, and proteins to the central nervous system. Liu et al. [65] identified the blood-brain barrier-permeable lipid BAMPA-O16B, which enables the co-delivery of CD47/PD-L1 siRNA, thereby downregulating immune checkpoints in glioma cells.

Other delivery methods that bypass the blood-brain barrier have further increased brain accumulation rates. Dong's research group developed lymph node-targeted LNPs and 5-HT3 receptor-mediated OS4T LNPs, which increased mRNA delivery to the brain by more than 50-fold and inhibited the progression of glioblastoma through IL-12 immunotherapy [149,187]. Combining blood-brain barrier penetration with precise targeting of pathological cells will further enhance the precision of treatment [188]. Mingzhu Gao [189] developed MLNPs for M2 microglia-targeted IL-10 mRNA delivery in ischemic stroke, promoting neuroprotective polarization. These advances collectively underscore the synergy between BBB penetration strategies and pathology-informed cell specificity.

Although breakthroughs have been made in the dual strategy of blood-brain barrier penetration and pathological targeting, current brain-targeted LNPs still have significant limitations. Most platforms exhibit limited specificity for the lesion and are prone to causing widespread transduction of extraneural targets. The dynamic remodeling of the blood-brain barrier across different pathological stages complicates achieving stable delivery performance, while the long-term risks of neurotoxicity and neuroinflammation associated with repeated dosing remain unclear, limiting clinical progress.

#### 4.1.4 Targeted delivery therapy for the heart

As the central circulatory organ, the heart maintains oxygen and nutrient perfusion throughout the body; therefore, nucleic acid delivery to the heart is crucial for the treatment of refractory cardiovascular diseases [[190], [191], [192]].

Using a rabbit model, Handa et al. [152] compared intra-coronary (IC), intravenous, and intramyocardial LNP delivery routes and demonstrated that intra-coronary administration achieves the most extensive cardiac distribution. VEGF mRNA-LNP delivered via the coronary artery route effectively reduced the infarct size and improved cardiac function (Fig. 11D).

Other optimized LNPs can also achieve precise cardiac intervention: ApoE-dependent cLNPs can mediate cardiac SERCA2A gene silencing [193]; VEGF-C mRNA-LNP promotes post-MI lymphangiogenesis [191]; acid-degradable PEG-LNP enhances cardiac gene editing efficiency [194]; ECM-conjugated LNP alleviates myocardial fibrosis by targeting EGFR siRNA delivery [195]; and ischemia-reperfusion-targeted LNP preferentially transfects cardiac fibroblasts to achieve local regulation [196].

Overall, myocardial-targeted LNPs offer highly promising tools for precision cardiovascular therapy. Nevertheless, it is important to distinguish between two distinct delivery paradigms: coronary-specific delivery via intracoronary injection represents a route-dependent organ enrichment strategy rather than systemically programmable targeting mediated by LNP biological identity. In contrast, true extrahepatic targeting via systemic administration—as achieved through lipid composition optimization or protein corona engineering for lung, spleen, or bone delivery—remains far more challenging for cardiac applications. Currently, systemic targeting efficiency to the heart remains low, and most cardiac delivery systems still rely on local or invasive routes. Furthermore, the dose-dependent cardiotoxicity and poor retention kinetics of existing LNPs hinder their practical clinical application. Beyond delivery carriers, the identification of novel disease targets is equally critical. For instance, sirtuin 6 has recently been identified as a promising therapeutic target for mitigating thoracic aortic aneurysm progression by maintaining mitochondrial homeostasis in vascular smooth muscle cells [197].

#### 4.1.5 Targeted delivery therapy for the kidneys

In acute kidney injury, LNPs loaded with SOD2 mRNA effectively restore mitochondrial redox homeostasis in an ischemia-reperfusion injury model, reduce the accumulation of reactive oxygen species (ROS) in the kidneys, and improve renal histological and functional recovery [198]. For ccRCC, well-designed LNPs can recruit circulating Vtn to form a specific protein corona that binds to the αvβ_3_ integrin overexpressed on ccRCC cells. This strategy increased mRNA delivery efficiency 952-fold in vitro and 42-fold in vivo, providing a novel corona-mediated targeting strategy for renal tumor therapy (Fig. 11E) [107].

Nevertheless, research on kidney-targeted LNPs remains in its early stages. Most studies focus on short-term functional recovery and lack long-term assessments of renal accumulation, toxicity, and chronic inflammatory responses. Meanwhile, the precise regulatory mechanisms underlying cap-mediated kidney targeting remain at the empirical level, and there is a lack of universal design guidelines.

#### 4.1.6 Targeted delivery for bone therapy

LNPs that target bone and cartilage have been developed for the treatment of musculoskeletal disorders. Yoon et al. [171] designed ionizable lipids functionalized with diphosphate groups that exhibit high affinity for hydroxyapatite, enabling specific bone targeting following systemic administration (Fig. 11F). Yu et al. [199] constructed CAQK-modified cartilage-targeted LNPs for the delivery of IGF-1 mRNA; this system enhances joint permeability, prolongs local retention time, reverses chondrocyte apoptosis, and promotes cartilage repair. Basha et al. [200] optimized PEG-modified bone-targeted LNPs to regulate GNAS expression, promote osteoblast differentiation, and treat osteoporosis.

In addition, we have compiled a list of LNP/RNA-based drugs that have already been approved for clinical use (Table 3) and those currently undergoing clinical trials (Table 4); all of these demonstrate the immense potential of LNP as a delivery vehicle.

**Table 3:** LNP/RNA drugs currently approved for market release.

Drug Name	Indications/Uses	Formulation	Administration	Type	Dose	Ref.
ONPATTRO	Adult-onset hereditary transthyretin-mediated amyloidosis causing polyneuropathy	solution	intravenous injection	siRNA	0.3 mg/kg,every three weeks	[96]
Comirnaty	COVID-19 vaccine	suspension	Intramuscular injection	mRNA	30 μg, twice, at 21-day intervals	[201]
Spikevax	COVID-19 vaccine	suspension	Intramuscular injection	mRNA	100 μg (0.5 mL), two doses, 28 days apart	[202]
mRNA-1345	Respiratory syncytial virus infection	suspension	Intramuscular injection	mRNA	50 μg (0.5 mL), single dose	[203]
mRNA-1283	COVID-19 vaccine	suspension	Intramuscular injection	mRNA	50 μg (0.5 mL), single dose	[204]

**Table 4:** LNP drugs currently in clinical research.

Drug Name	Indications/Uses	Administration	Active ingredient	Clinical stage	Ref.
NTLA-2001	Amyloidosis caused by transthyretin (ATTR)	intravenous injection	CRISPR-Cas9 mRNA	Phase III	[205]
IL-22BP/LNP	Refractory malignant solid tumors	intratumoral injection	IL-22BP mRNA	Phase I	[206]
mRNA-3927	Propionic acidaemia	intravenous injection	PCCA/PCCB mRNA	Phase I/Phase II	[207]
MRT5005	Pulmonary dysfunction in cystic fibrosis	Nebuliser inhalation	CFTR mRNA	Phase I/Phase II	[208]
mRNA-1893	Prevention of Zika Virus	Intramuscular injection	prM-E mRNA	Phase II	[209]
CV7202	Rabies Prevention	Intramuscular injection	Rabies virus G protein (RABV-G) mRNA	Phase I	[210]
QTP104	Prevention of SARS-CoV-2 (COVID-19) infection	Intramuscular injection	RepRNA encoding the SARS-CoV-2 spike protein	Phase I	[211]
BEAM-302	Alpha-1 Antitrypsin Deficiency (AATD)	Intravenous injection		Phase I/Phase II	[212]
mRNA-2752	Advanced solid malignant tumors	Intratumoural injection	OX40L、IL-23、IL-36γ mRNA	Phase I	[213]
HDT-301	Prevention of COVID-19 (infection caused by SARS-CoV-2)	Intramuscular injection	RepRNA encoding the SARS-CoV-2 Spike protein	Phase I	[214]
ARCT-810	ornithine transcarbamylase(OTC)deficiency	Intravenous injection	OTC-mRNA	Phase II	[215]
SYS6017	Shingles	intramuscular injection	Encoding VZV glycoprotein E (gE) mRNA	Phase I	[216]
DCR-MYC	Hepatocellular carcinoma	Intravenous injection	Small interfering RNA (siRNA) targeting the MYC oncogene	Phase I/II (discontinued)	[217]
NTLA-2002	Hereditary angioedema type I or type II	Intravenous injection	Cas9-mRNA + KLKB1-specific gRNA	Phase III	[218]
JCXH-211	glioma	Intratumoural injection	The srRNA encoding the IL-12 protein	Phase I	[219]
CD-801	cholangiocarcinoma	Arterial injection	SaRNA encoding hepatic nuclear factor 4α (HNF4α)	Phase I	[220]
ARCT-032	Cystic fibrosis	Nebuliser inhalation	CFTR mRNA	Phase II	[221]

#### 4.1.7 Targeted delivery therapy for the pancreas

Beyond the traditional extrahepatic organs, the pancreas has emerged as a challenging but highly attractive target for LNP-mediated RNA delivery, particularly for the treatment of pancreatic ductal adenocarcinoma and diabetes. Recent breakthroughs have demonstrated the feasibility of systemic LNP delivery to pancreatic tissues. Lei et al. [222] identified a universal principle for pancreatic-selective delivery based on organ capsule filtration and developed AH-LNP, which undergoes size enlargement after protein assembly to facilitate capsule-filter-mediated retention and efficient genome editing via Cas9 mRNA/sgRNA delivery, with safety and translational potential validated in non-human primates. In one notable advance, the endogenous targeting (ENDO) platform was developed to achieve systemic mRNA delivery to the pancreas by recruiting VDR from plasma to form a unique protein corona, enabling precise β-cell-specific gene editing and protein replacement [95]. In parallel, pancreas-targeted LNPs have been designed to deliver interleukin-12 mRNA for relatively non-invasive therapy against orthotopic pancreatic ductal adenocarcinoma, achieving local immunomodulation and tumor suppression while minimizing systemic toxicity [223]. Complementing these direct targeting strategies, an endocrine-driven approach utilizing liver-targeted lipopolymer nanoparticles for FGF21 mRNA delivery has shown consistent therapeutic benefit in experimental acute pancreatitis models [224] These complementary strategies collectively underscore the growing potential of LNP-mediated RNA therapeutics for pancreatic diseases, a class of conditions that have historically been refractory to conventional drug delivery approaches.

### 4.2 Bottlenecks in clinical translation

Despite significant advances in extrahepatic LNP delivery, several translational bottlenecks remain—ranging from differences in protein corona formation across species and loss of targeting efficacy upon repeated administration, to manufacturing challenges and immunogenicity concerns. Collectively, these obstacles impede the clinical translation of tissue-specific LNPs.

#### 4.2.1 The species barrier in protein corona evolution

Most preclinical studies of LNP-mediated extrahepatic targeting rely on mouse models, but plasma protein profiles differ not only between species but also across strains, sexes, and age groups within the same species. For example, the plasma concentrations of key opsonins, including ApoE, Vtn, and β_2_-GPI, vary considerably between C57BL/6 and BALB/c mice, between males and females, and between young and aged animals [[225], [226], [227], [228]].

Since these biomolecules directly form the LNP protein corona and thus affect subsequent tissue targeting, even a small variation in their relative amounts can alter the route of biodistribution and result in non-uniformity of targeting efficiency among different laboratories or experimental groups. The interspecies barrier will be more pronounced in clinical applications of these studies. Surface Plasmon Resonance studies have shown that ApoE and its orthologues from various species have significantly different binding strengths and kinetic characteristics, thus resulting in structural variations in the corona among preclinical animal models and human patients [229]. Due to the different targeting mechanisms in different species, the targeting strategies based on rodent research may not be directly transferable to humans, and thus the initial clinical benefits of extrahepatic LNP technology are reduced. To reduce the risk of translational failure, models closer to humans, such as humanised mouse systems or validation in non-human primates, should be employed, and standardisation of the plasma material used in corona characterization studies is required [230]. Glucocorticoid pre-treatment can now be employed to address the problems mentioned above by suppressing macrophage-mediated clearance temporarily and thus increasing the transport efficiency of LNP-mRNA and the effect of genome editing [231].

The environment of the biological fluid may also affect the formation of the corona. A study comparing LNPs incubated in human plasma versus those in cerebrospinal fluid (CSF) showed that, despite the plasma having over 100 times more total protein, coronas formed in the CSF had a relatively high proportion of ApoE (1.08% of total adsorbed protein compared to only 0.075% in plasma), which is due to the different apolipoprotein composition of CSF. This enrichment can help increase the uptake of nanoparticles by nerve cells [91].

#### 4.2.2 Changes in tissue distribution following repeated dosing

Chronic RNA therapy requiring repeated dosing faces the ABC phenomenon, driven by anti-PEG IgM antibodies that form immune complexes with subsequently administered LNPs [232]. Mechanistically, the magnitude of ABC depends critically on PEG-lipid design: C14-PEG lipid dissociates from LNP surfaces at ∼45%/h, whereas C18-PEG lipid dissociates at only ∼0.2%/h, resulting in prolonged surface exposure and 2.3-fold higher anti-PEG IgM titers for C18 formulations [233]. Importantly, ABC occurs regardless of administration route (intravenous, intramuscular, or intraperitoneal) [234] and is not alleviated by altering PEG-lipid type or molar ratio (1.0–2.5 mol%), with efficacy loss correlating directly with elevated anti-PEG IgM levels [235].

In addition, after several rounds of LNPs, the innate immune system is activated; therefore, these nanoparticles are rapidly cleared, and antibodies against polyethylene glycol are generated. The resulting immune complexes are further removed from the body systemically and do not accumulate in the liver [236,237]. Research has demonstrated that degradable PEGylated lipid nanoparticles lessen nonspecific immunological stimulation and mitigate accelerated blood clearance effects, thereby enhancing the consistency of outcomes during repeated administration [238]. Researchers have found that using Y-shaped PEGylated LNPs can significantly reduce ABC efflux rates, thereby improving cancer treatment outcomes [239]. Current optimization strategies have had limited impact on this issue. The long-term effects of repeated LNP administration, particularly immune memory, macrophage reprogramming, and microenvironmental changes, remain unclear. Moreover, when administering repeated injections, there is a lack of standardized guidelines on dosage and formulation to ensure reaching extrahepatic targets.

#### 4.2.3 Effects of the freeze-drying process on the physical properties of LNPs

The physical properties of LNP (such as particle size, polydispersity index, zeta potential, and RNA encapsulation efficiency) largely determine its performance in terms of extrahepatic targeting and in vivo stability. The main challenge faced by the mRNA LNP platform is its reliance on the cold chain to maintain stability during delivery and for long-term storage [240]. Although lyophilization is regarded as a key approach for resolving the long - term storage issue of LNPs [241], the freezing and dehydration processes can exert mechanical stress on the LNP structure, resulting in aggregation [242] or even RNA leakage [243], thus undermining their physical properties and targeting capabilities.

The impacts of the lyophilization process on the physical properties of LNPs are mainly reflected in three aspects. Firstly, low temperature crystallization and dehydration during lyophilization, especially in the absence of a protective agent, can induce the aggregation of LNP particles. This aggregation results in an increase in particle size, a rise in the PDI, and a decrease in LNP uniformity [244,245]. Nevertheless, extrahepatic targeted delivery (e.g., to the lungs or tumors) places more stringent requirements on LNP particle size. Overly large particles may impede LNPs from penetrating target tissue barriers (such as the tumor vascular endothelial barrier) and render them more prone to clearance by the MPS [246]. Secondly, during the freeze-drying process, the lipid bilayer structure within the LNP is disrupted, leading to a decline in RNA encapsulation efficiency. Free RNA is readily degraded by endogenous nucleases, and the efficiency of LNP escape from endosomes is diminished, thus influencing the intracellular delivery of RNA. Even with optimized lyophilization protocols, long-term storage still leads to slight particle enlargement and reduced encapsulation efficiency, disproportionately impairing fine-tissue targeting performance [247].

Current optimization strategies, including the addition of cryoprotectants [248], buffer adjustment [249], replacement of lipid components [250], can effectively mitigate structural damage to LNPs and maintain transfection activity. Furthermore, recent studies have explored the use of free PEG as a cryoprotectant: LNPs in PEG media (molecular weight 400–6000 Da) remained stable for 4 weeks at −20 °C with minimal changes in particle size; however, none of the PEG formulations tested were able to prevent aggregation during freeze-drying, as dehydration severely disrupts the mechanisms stabilizing the electric double layer. Based on the above, it can be inferred that freeze-drying-induced damage is closely related to formulation, and optimization should be carried out for specific cases [251].

#### 4.2.4 Other clinical translation bottlenecks

Existing studies have systematically reviewed non-viral delivery systems applied in cancer gene therapy [252]. LNPs for extrahepatic delivery face several intrinsic drawbacks that hamper clinical use. The blood–brain and blood–lung barriers are major obstacles, severely restricting tissue penetration, and existing ligand functionalization approaches fail to provide reliable targeting to these remote regions [253]. While LNP platforms possess weaker immunostimulatory potential than viral delivery systems, they are capable of activating toll-like receptor 4-mediated innate immune responses in a dose- and administration frequency-dependent manner [254]. Immunostimulation may provoke localized inflammatory reactions and cytokine release within peripheral tissues such as the pulmonary system and splenic organ, while the positively charged lipids contained in these LNPs can additionally exacerbate intrinsic cytotoxicity at the cellular level [255].

Another problem in the industrialization of manufacturing for lipid nanoparticle therapy is the scale-up challenge [256]. The construction of LNPs is very sensitive to changes in the production environment, and thus, batch-to-batch variations in product quality have occurred that prevent commercialization. Microfluidic-based fabrication is required for the production of high-quality LNPs, but at an increased scale of production, there are problems with maintaining a uniform mixing environment and a monodisperse size distribution. This technical instability significantly reduces the reproducibility of their activity when delivered to tissues for extrahepatic administration [257].

Furthermore, the administration of multiple doses creates additional immunogenic barriers to long-term therapy. Stealth-modified LNPs carrying a PEG chain can repeatedly trigger the generation of anti-PEG endogenous antibodies after multiple injections, thereby activating the ABC process [236,258]. This immune response drastically lowers nanoparticle accumulation in peripheral target tissues and potentially induces pseudo-allergic adverse reactions. Even without detectable anti-PEG immune factors, iterative LNP delivery is able to reshape the biological functions of tissue-resident macrophages and hepatic Kupffer cells, ultimately suppressing the intracellular expression efficiency of delivered mRNA payloads [259].

Stable manufacturing output remains a core technical barrier for the industrialization of extrahepatic-targeted LNPs. Microfluidic preparation technology can generate nanoparticles with controllable diameters ranging from 70 to 190 nm with excellent repeatability at laboratory scale. Nevertheless, scaling up production capacity to over 100 L for commercial use leads to uneven fluid mixing and unstable shear force conditions [260]. Given the high susceptibility of LNP properties to flow velocity, flow proportion and ambient temperature, systematic process analytical technology and quality-by-design manufacturing modes are essential to guarantee consistent batch quality [261].

To address these shortcomings, various technical optimization strategies have been proposed, such as developing biodegradable lipid scaffolds, designing cleavable stealth surface modifications, and optimizing clinical dosing regimens. However, each improvement strategy can only address individual technical shortcomings in current LNP systems. Currently, there is no systematic and comprehensive design approach capable of simultaneously overcoming the risks of immunostimulation and unstable manufacturing performance. This technical gap constitutes the primary bottleneck limiting the widespread clinical application of the LNP platform for extrahepatic targeted delivery.

## 5 Summary and prospect

Research on RNA-LNP is moving beyond its natural accumulation in the liver toward programmable nanoparticle biological identity. To achieve off-organ targeting, integrated control of lipid structure, physicochemical properties, and protein corona is required. Three strategies support this goal: diverse lipid backbones, multiparameter particle control, and reconfiguring the corona using SORT and POST platforms. To avoid indiscriminate screening, the following concise and practical formulation criteria can be distilled from the literature. For lipids, degradable ionizable compounds with a pKa of 6.2–7.0, DSPC-assisted lipids, and plant/vitamin D sterols can reduce ApoE binding; meanwhile, the addition of C14 PEG lipids should be limited to 1.0–2.0 mol% to mitigate the ABC reaction. LNPs should be optimized to 70–120 nm, and their tissue-specific ζ-potential and pKa values should be adjusted to balance circulation and endosomal escape. SORT lipids or 6R/6D peptides can recruit Vtn in the lungs or in the spleen. Formulations containing ≤5 lipid components feature LNPs with targeted affinity and are suitable for PAT/QbD microfluidic production and lyophilization processes.

There are significant differences in drug delivery efficacy across different organs: LNPs that can target the lungs and spleen can reduce off-target effects in the liver by more than 80%, but transport to the brain is severely limited by the BBB. Formulations targeting the kidneys, heart, and bones remain underdeveloped. Multiple translational barriers currently exist: immune reactions against PEG limit repeated dosing, although cleavable PEG offers a solution. Ionizable lipids can trigger TLR-mediated inflammation, and the structure–corona–function relationship remains unclear, requiring elucidation through AI modeling and proteomic analysis. Interspecies proteomic differences hinder clinical translation, which can be addressed using organ-on-a-chip platforms and nonhuman primate studies. Future research will integrate proteomics with computational tools to develop predictive design frameworks and reduce animal testing. Overcoming persistent immune memory and tissue remodeling challenges will enable long-term RNA therapies, advancing nanomedicine from empirical formulation toward rational nanoparticle programming for systemic disease treatment.

In short, the next ten years will see a new development in RNA therapeutics: moving away from LNP designs that rely solely on physicochemical rules, they will be programmed to achieve specific biological effects. To achieve this goal, one needs to understand how the engineered material interacts dynamically with the body's internal environment better, and then use RNA-based treatment technologies more flexibly and precisely for systemic diseases.

## CRediT authorship contribution statement

**Yu Liu:** Investigation, Methodology, Supervision, Writing – original draft, Writing – review & editing. **Xiao Guo:** Investigation, Methodology, Supervision, Writing – original draft, Writing – review & editing. **Qin Hu:** Investigation, Methodology, Writing – review & editing. **Chunyuan Gan:** Investigation, Methodology, Writing – review & editing. **Shili Nie:** Investigation, Methodology, Writing – original draft. **Jingheng Xiang:** Investigation, Methodology, Writing – original draft. **Yao Liu:** Investigation, Methodology, Writing – original draft. **Jie Zou:** Investigation, Methodology, Writing – original draft. **Xintong Wu:** Conceptualization, Funding acquisition, Project administration, Supervision, Writing – review & editing. **Chong Li:** Project administration, Supervision, Writing – review & editing. **Yaqin Tang:** Conceptualization, Funding acquisition, Investigation, Methodology, Supervision, Writing – original draft, Writing – review & editing.

## Declaration of competing interests

The authors declare that they have no known competing financial interests or personal relationships that could have appeared to influence the work reported in this paper.

## Data Availability

No data was used for the research described in the article.
